# Analyzing climate change impacts on health, energy, water resources, and biodiversity sectors for effective climate change policy in South Korea

**DOI:** 10.1038/s41598-021-97108-7

**Published:** 2021-09-16

**Authors:** Tae Hoon Moon, Yeora Chae, Dong-Sung Lee, Dong-Hwan Kim, Hyun-gyu Kim

**Affiliations:** 1 College of Social Science, Department of Urban and Real Estate, Departmenet of Public Administration, Chung Ang University, 84 Heukseok-ro, Dongjak-gu, Seoul, 06974 South Korea; 2 Korea Environment Institute, Bldg B, 370 Sicheong-daero, Sejong, 30147 Republic of Korea; 3 Spatial Information Industry Promotion Agency, 242 Pangyo-ro, Bundang-gu, Seongnam-si, 13487 Gyeonggi-do Korea

**Keywords:** Climate-change adaptation, Climate-change impacts

## Abstract

This study analyzes how climate change affects the economy, society, and environment in South Korea. Then, the study explores the ways to strengthen capabilities that can alleviate climate change impacts. To find them, the study employs a system dynamics simulation method and builds a model with several sectors including the urban, rural, population, and social-environmental sectors. The study compares the size of climate change damages in rural and urban areas. The results with representative concentration path (RCP) 8.5 show that the size of climate change damage will continue to increase by 2050. The projected damages from the reduced industrial outputs in urban areas will be larger than that in rural areas. The results also show that the service sector will face stronger impacts from climate change than the manufacturing and agricultural sectors. However, the total size of damage in the rural areas will be bigger than that of the urban areas. It is because the size of reduced industrial outputs per capita in the rural areas is twice bigger than that of the urban areas. The climate change damage in the social and environmental sectors (including a loss of biodiversity and an increase in health costs) account for the largest part of the total damage. The study finally provides suggestions and policies that can improve the capabilities to reduce the climate change damages. One of the major suggestions of this study is that the increase in the climate change budget corresponding to the GDP growth can minimize the size of climate change impacts.

## Introduction

In 2015, the United Nations adopted 17 Sustainable Development Goals (UN SDGs), which replaced the Millennium Development Goals (MDGs), with the target of achieving these goals by the year 2030. The 17 SDGs can further be categorized into the 5Ps: people, prosperity, planet, peace and institution, and partnership. The bulk of the SDGs consists of goals related to people: ending poverty and hunger, ensuring health and well-being, providing quality education, and improving gender equality. By realizing these goals, we can achieve “development as freedom,” which enhances people’s capacity to do what they want to do—something that Amartya Sen defined as the ultimate goal of development^1^. To attain this ultimate goal, we need to work toward achieving two Ps simultaneously—conserving the planet and securing sustainable prosperity—while also ensuring a peaceful, inclusive society and institutions, as well as global partnership. To save our planet, the most urgent goal is mitigating climate change and preparing for it. According to the 4th climate report from the International Panel on Climate Change (IPCC), we have only a small amount of time left to mitigate the impact of climate change before the opportunity window closes. To keep the global temperature increase within 2℃, we need to limit total accumulated emissions of CO2 equivalent (CO2eq) greenhouse gases (GHGs) to within 2900 Gigaton (Gt) CO2eq. However, we have already released 1900 Gt eqCO22. Since we have been releasing about 50 Gt CO2eq annually as of 2010, we have only 20 years from 2010 before we shut down total GHG emission to zero by the year around 2030, which is the target year of UN SDGs. In light of the climate crisis as well as the rapid extinction of biodiversity globally, this study analyzes the impact of climate change on various aspects of South Korean society and explores policy alternatives to mitigate this impact.

The impact of climate change in Korea is growing rapidly, mainly due to typhoons, heavy rains, droughts, cold waves and abnormal temperatures. According to the 2020 Abnormal Climate Report published by the Korea Meteorological Administration^2^, the number of property damage and casualties was 1.2685 trillion won(about 1.153billion USD, 1 USD is equivalent to about 1,100 KRW) and 46 lives due to typhoons and heavy rains in 2020, tripled from the average annual damage (388.8 billion won property, 14 lives) in the past decade. In addition, 6,175 landslides (1,343 hectare, ha) occurred, the third-largest number in history since 1976, and more damage (123,930 ha) occurred during the crop harvest season than in 2019 (74,165 ha). And, typhoon Maisak caused a power outage in 294,818 houses, nearly double the number of Typhoon Lingling (161,646 houses) in 2019. Winter abnormal temperatures occurred nationwide, and winter temperatures in January were the warmest since 1973, caused many summer-insects(flower cicadas, walkingsticks, leaf-eating insects etc.) and 6,183 hectares of forest damage nationwide.

For the study purpose in this context, the study employs a system dynamics simulation method to build a model. The model can analyze the impact of climate change on the environmental, economic, and social areas while considering the dynamic interaction among these areas. The system dynamics is a framework for understanding and managing complex problems that are highly interrelated like climate change. With the application of system dynamics, the study can capture the dynamic changes in different sectors over time driven by climate change and how the different sectors affect each other. The simulation model was built for urban and rural areas to explore customized policy alternatives for each area. Industry, energy, water resource, population, and environment were included in the model to estimate the impact of climate change on each of these sectors and to analyze the overall impact on South Korean society. The simulation period was set from 2000 to 2050.

The literature review focused mainly on studies that investigated the impact of the climate crisis via simulation analysis. In this respect, we carefully reviewed Forrester’s^3^ *World Dynamics*; the capital sector in the World3 model—the base model of Meadows et al.’s^4^ book, *Limits to Growth;* Meadows et el.’s^5^ *The Dynamics of Growth in a Finite World;* and several updates versions of *Limits to Growth* ^6–8^. The capital sector of the World3 model is important for this study because it provides important insights into the circular causation structure of climate-change-induced impact: climate change impact → decrease in agricultural and industrial productivity → decrease in industrial output → decrease in government budget for climate change → increase in climate-change-induced damage cost → further decrease in agricultural and industrial productivity. A critical study examines Nordhaus’ dynamic integrated climate change (DICE) model with a revised conclusion and argues that the DICE model underestimated the feedback effects of temperature change and carbon storage capacity on the carbon cycle^9^. Other studies compare climate change policy alternatives^10^, examine climate change impacts on water supply and demand in Mexico^11^, and develop the C-ROADS climate policy model^12^. Related studies in the South Korean context investigate the impact of carbon tax on energy, economy, and the environment^13^, examine the management of water supply facilities in response to climate change^14^, conduct policy evaluation on greenhouse gas mitigation^15^, and estimate the impact of sea-level rise on flooding, using system dynamics and a geographic information system link model^16^. However, despite several such studies on different areas of climate change, research on the overall impact of climate change on the South Korean economy, society, and environment is limited.

Chae et al.^17^ estimate the economic impacts of climate change using a top-down policy analysis of the greenhouse effect model as well as bottom-up models in the fields of health, agriculture, water resource, ecosystem, and sea-level rise in South Korea until 2100. However, they do not consider the propagation and feedback effects of the impacts on other sectors.

Governments have put such efforts to protect climate change by implementing various policies that can lower greenhouse gas (GHG) emissions or promote the ability to adapt to climate change. Implementing those policies, however, require the government’s budget. And, it is important to find out whether this government’s budget increases are effective to alleviate the climate change damages. One recent study closely related to this paper investigated the government’s policy response to mitigate climate-change-induced risk by utilizing the climate change risk model^18^. The study shows that the increased government budget could effectively lower the size of climate change damage. In addition, the study demonstrates that an earlier budget increase could be more effective than a delayed budget increase. The global organization also has closely looked at the effectiveness of climate-related public policy in various sectors. The Organization for Economic Co-operation and Development (OECD) regularly publishes the Environmental Outlook. In this study, the OECD investigates different climate policies implemented in biodiversity, water, and human health and then estimates their effectiveness. According to the OECD Environmental Outlook to 2050^19^, without any action, the terrestrial biodiversity is projected to decrease more than 10% by 2050. However, with the full implementation of policies in biodiversity, the decrease can be slowed down. The current study expands on the previous research by estimating the long-term climate change impact on various sectors of the South Korean society, which has not been exhaustively analyzed by prior studies. By including the social, environmental, energy, and water sectors, this study explores the climate change impact, identifies policy measures from a broad perspective, and explores diverse policy alternatives to mitigate the expected impacts of climate change.

## Research method

The system dynamics method is useful to analyze complex problems that involve highly interrelated/interactive factors and circular feedback structures. Two key components of system dynamics are circular feedback thinking and system dynamics simulation. Circular feedback thinking is a framework for understanding certain problems or phenomena from a dynamic and circular feedback causation perspective. This framework is used to translate problematic phenomena into a computer simulation model using system dynamics simulation software. The simulation results reveal dynamic changes in the variables of interest over time, allowing the researcher to experiment with various policy alternatives to mitigate problems^18,20,21^.

**Figure 1:**
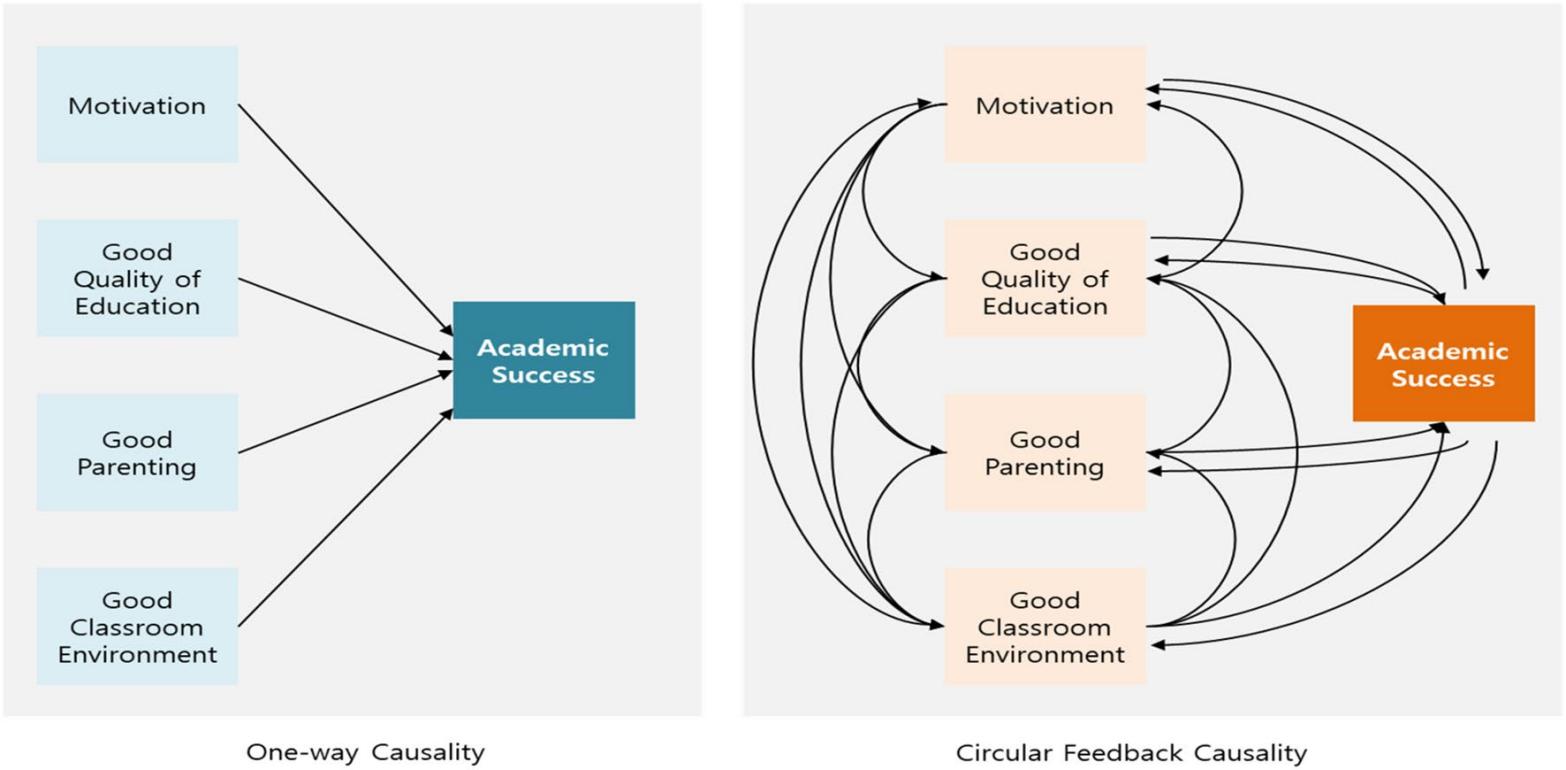
One way causality vs. Circular feedback causality^22^.

In general, the most intuitive and universal way to understand phenomena is correlational understanding. As we experience and observe several phenomena, we learn the correlations between them and observe, understand, and explain the world based on these correlations. However, the phenomenon of correlation understanding is often static. In contrast, system dynamics understands phenomena as dynamic changes over time. The difference between a static understanding of the phenomenon and a dynamic and cyclical understanding can be illustrated by Fig. 1. The figure is based on the idea of Richmond and Peterson^22^. For example, if the factors that determine academic success are motivation, attendance, quality of education, parental interest, intelligence, health, and classroom environment, the static approach measures the impact of each of these factors on academic success. It is assumed that each independent variable is independent of each other. In practice, however, independent variables interact with each other, and independent and dependent variables also affect each other. Higher quality of education can motivate students, which can improve their academic performance so that they can attend harder, with higher motivations. Encouraged by this, teachers can teach harder and parents can become more interested in improving the classroom environment and increasing students' motivation. In Fig. 1, the left side of thinking can be called static, short-line, and partial thinking, and the right side of thinking can be called dynamic, cyclical, and integrated thinking, and it is the Causal Loop Diagram (CLD) that depicts the cyclical, circular feedback causation and dynamic structure of variables.

Causal Loop Diagram (CLD) is a representation of the relationship between multiple variables that causes the problem or phenomenon we are trying to solve in a series of circular causation structures. As general statistics understand, the relationship between independent and dependent variables is not seen as the cause and effect of one-way relationship, but as the circular causality such as independent variables cause dependent variables and cause independent variables circularly. It is understood as a circular feedback relationship in which variable X influences variable Y and the affected Y influences X again. Therefore, the distinction between independent and dependent variables is not necessary. It is called system dynamics because study focus is to see changes of variables over time in a system that is consisted of interrelated and interacting variables. In short, system dynamics is a perspective and framework for understanding and explaining phenomena from the perspective of circular feedback causality, and a methodology for modelling and analyzing phenomena based on this understanding^18,23^.

A system with a positive feedback loop in a causal map can function as a virtuous cycle or a vicious cycle because the variable of interest is characterized by exponential growth or decline. If there is a strong promotional intervention at major policy intervention points, the system will rise to a virtuous cycle, but if the intervention is lowered, it will return to a vicious cycle in which the performance of the total system will continue to decline. If the trigger of policy intervention begins to create a virtuous cycle of the system, the system will go into a continuous virtuous cycle, but in the opposite case, it should be noted that appropriate policy intervention points and moderate-scale intervention can be important issues. System dynamics is not focused on a system, but rather on a problem that is handled from a dynamic and feedback perspective^21,24,25^. System dynamics modeling involves several stages of problem identification and definition, system conceptualization, model formulation, analysis of model behavior, model evaluation, and policy analysis. At the system conceptualization stage, which variables to include or exclude is determined. This process of defining the system boundaries is closely related to that of problem definition. Only those variables that interact with and are directly related to the defined problem are included in the model for simulation, and other variables external to the system variables are treated as exogenous variables. Relationships among variables included in the model are structured as a circular feedback causation process, and this process is usually based on a literature review, interviews, and observation by practitioners. In this study, a system dynamics model is constructed with a closed-loop causality perspective on problem definition, which allows for dynamic changes in the variables of interest over time and improves the performance of various types of policy tests^20^.

## Building the model

**Figure 2:**
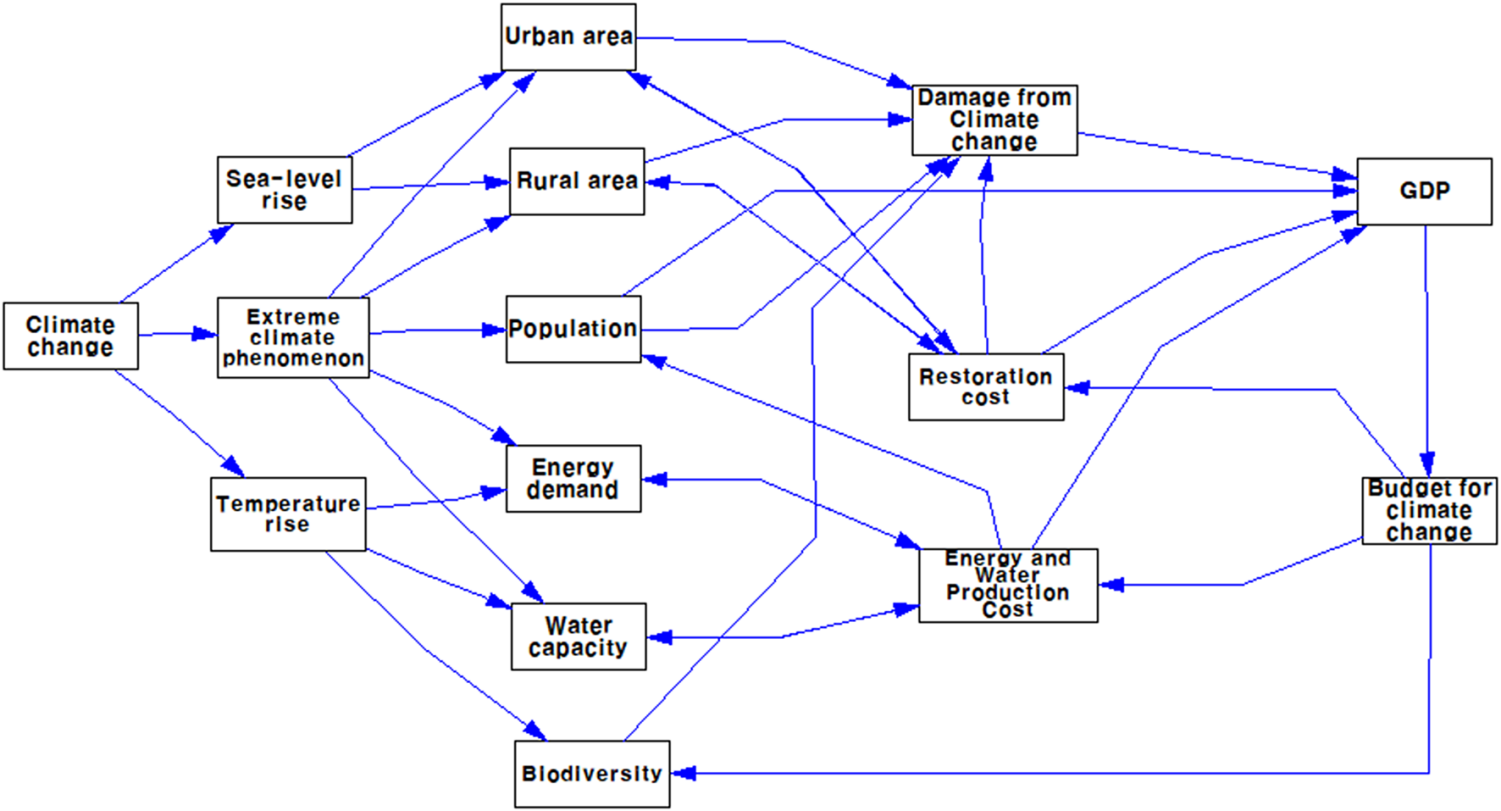
Structure of the climate change impact model. GDP = Gross Domestic Product.

A simplified structure of the climate change impact model is depicted in Fig. 2. In this model, the climate change impact is structured as coming from three sources: sea-level rise, extreme climate phenomena, and temperature rise. All or some of these factors affect several selected areas in this model, such as urban and rural areas, population, energy, water supply and demand, and biodiversity. The climate change impact on these areas incurs a direct damage cost, restoration cost, and cost for response preparedness. Increasing the total cost incurred by climate change negatively affects the national economy and gross domestic product (GDP). This, in turn, affects the government budget expenditure for climate change response.

**Figure 3:**
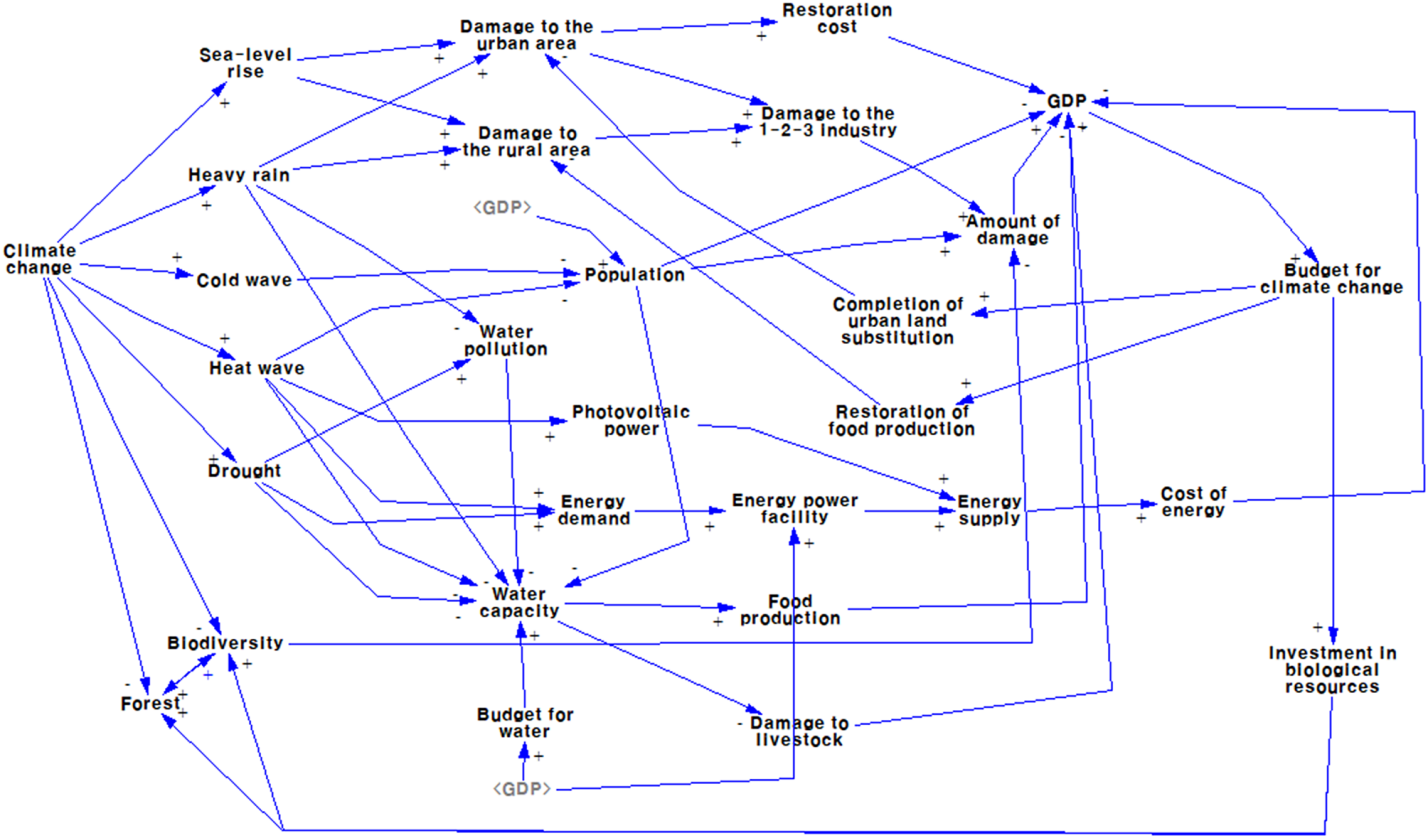
Causal-loop diagram of the climate change impact model. “1–2-3 industries” refers to the primary, secondary, and tertiary industries.

The conceptual structure of the climate change impact model can be translated into a causal-loop diagram, as shown in Fig. 3. Figure 2 shows overall structure of the model built and used for simulation in this study. Linear relationship between sea-level rise and urban and rural area indicates impact of sea level rise on urban and rural area near the sea, where part of areas can be flooded due to sea level rise. The causal-loop diagram shows the interactive and interrelated variables in a circular way, which can help investigate the problematic behavior of the system. The diagram depicts the relationships among the variables and the serial, interactive, and circular effects of climate change on urban and rural areas, population, water, energy, biodiversity, forest, national economy, GDP, and government budget use and investment for climate change response. Plus + , and negative—sign on arrowhead indicate relationship between the two variables. When two variables move to the same direction (increase or decrease), + sign is attached on the arrowhead. But, when the two variables move to the opposite direction (one increase and the other decrease, or vice versa),—sign is used on the arrow. For example, the further climate change, the more days of cold wave and heat wave are expected to occur. Two variables move to the same direction and thus, + sign is used. However, the further climate change, the less biodiversity will be resulted. Since the two variables move to the opposite direction,—sign is used on the arrowhead. This is important to understand system behavior over time. A loop with even number of negative signs on arrowhead is positive loop and odd number of negative signs on arrowhead is negative loop. A system with a positive feedback loop in a causal map can function as a virtuous cycle or a vicious cycle because the variable of interest is characterized by exponential growth or decline. If there is a strong promotional intervention at major policy intervention points, the system will rise to a virtuous cycle, but if the intervention is lowered, it will return to a vicious cycle in which the performance of the total system will continue to decline. If the trigger of policy intervention begins to create a virtuous cycle of the system, the system will go into a continuous virtuous cycle, but in the opposite case, it should be noted that appropriate policy intervention points and moderate-scale intervention can be important issues. This causal-loop diagram was translated into a stock-flow diagram for computer simulation. For modeling, simulation, and policy experiments, the Vensim DSS software for Windows Version 10 was used. The full set of flow diagrams for this model is presented in Appendix A.

## Simulation results

### Parameters

Data on the parameters used for model simulation were obtained from various sources, including central and local government statistics, reports from research institutes related to the sectors modeled in this study, and the Korea Adaptation Center for Climate Change. Variable names, parameter values, equations for individual parameters, and data sources are listed in Table 1.

**Table 1:** Selected model parameters, equations, statistics, and sources.

Sector	Variable	Parameter	Unit	Equations	Data Source
Urban	Urban area ratio	0.175	Ratio	Total urban area in South Korea/South Korea’s total land (2014)	Ministry of Land (www.mollt.go.kr)
	Development cost per unit of area	781.4	Billion won	Average construction cost of second new town^1^/Average area of second new town	Ministry of Land (www.mollt.go.kr)
	Ratio of 1–2-3 industries in the urban area	0.01, 0.37, and 0.61	Ratio	Average gross regional domestic product ratio of the primary, secondary, and tertiary industries in the urban area (2005 ~ 2013)	Statistics Korea (www.kosis.kr)
	Disaster restoration cost per unit of area	79.7	Billion won	Average flood damage assessment value of the urban area^2^/average flood-damaged urban area^3^ (2010 ~ 2013)	Ministry of Public Safety and Security (www.safekorea.go.kr)
Agriculture	Rural area ratio	-	Ratio	1—urban area ratio	Ministry of Land (www.mollt.go.kr)
	Restoration cost per unit of rural area	0.72	Billion won	Average flood damage assessment value of the rural area/average flood-damaged rural area. (2010 ~ 2013)	Ministry of Public Safety and Security (www.safekorea.go.kr)
	Reduction in productivity per unit of rural area	0.01	Ratio	According to research conducted by the Korea Rural Economic Institute, abnormal weather can reduce rice production by 1%	Korea Rural Economic Institute (2012), “Impacts and countermeasures of climate change on food supply in Korea”
	Reduction in productivity per heat day	0.06	Ratio	According to research conducted by the Korea Rural Economic Institute, high temperature can reduce rice production by 5.8%	Korea Rural Economic Institute (2012), “Impacts and countermeasures of climate change on food supply in Korea”
	Reduction in productivity per drought day	0.06	Ratio	According to research conducted by the Korea Rural Economic Institute, high temperature can reduce rice production by 5.8%	Korea Rural Economic Institute (2012), “Impacts and countermeasures of climate change on food supply in Korea”
	Food^4^	23,024,000,000	Kg	Food production—food consumption	Statistics Korea (www.kosis.kr)
	Cost per unit of food importation	4.9187e-007	Billion won	Average food importation cost (2007 ~ 2012)	Korea Customs Service (2013), “Grain import price soaring compared to last year”
Energy	Energy capacity	76,000	MW	Construction for energy capacity—aging of capacity	Statistics Korea (www.kosis.kr)
	Aging time	40	Year	Average design life of thermal power plants	Construction Economic (www.cnews.co.kr)
Water	Rainfall rate	-	Kg	1,230,000,000 * (urban area + rural area)	-
	Water demand per person	-	Kg	153,079 * (1 + effect of heat wave on water demand)	Ministry of Land (www.mollt.go.kr)
	Water demand per livestock	-	Kg	1,514.88 * (1 + effect of heat wave on water demand)	Ministry of Land (www.mollt.go.kr)
	Water demand per unit of arable land	8.95412e + 008	Kg	-	Ministry of Land (www.mollt.go.kr)
	Demand of industrial water	6.082e + 012	Kg	-	Ministry of Land (www.mollt.go.kr)
Population	Total fertility rate	-	Person	Estimate linear trend function using the total fertility rate data between 2000 and 2014 and extrapolate it to 2050	Statistics Korea (www.kosis.kr)
	Number of vulnerable people	-	Person	Population below 14 years + population older than 65 years	-
	Crude birth rate	-	Ratio	(Birth rate/population) * 1,000	-
	Crude death rate	-	Ratio	(Annual death rate/population) * 1,000	-
	Ratio of workforce	-	Ratio	[(Population aged 15–49 years + population aged 50–64 years)/total population] * 100	-
	Ratio of older aged population	-	Ratio	(Population older than 65 years/total population) * 100	-
Social and Environmental	RCP 8.5 temperature rise	-	°C	Build lookup function using the representative concentration pathway (RCP) 8.5 scenario (2000 = 11.3 °C, 2025 = 12.5 °C, and 2050 = 14.4 °C)	Temperature projected by the Korea Meteorological Administration (KMA) in 2012 under the RCP8.5 scenario KMA (2012), “Prospect of climate change in Korea peninsula”
	RCP 4.5 temperature rise	-	°C	Build lookup function using the RCP 4.5 scenario (2000 = 11.3 °C, 2025 = 12 °C, and 2050 = 13.6 °C)	Temperature projected by the KMA in 2012 under the RCP4.5 scenario Korea Meteorological Administration (2012), “Prospect of climate change in Korea peninsula”
	Malaria	-	Ratio	Build lookup function using the Korea Ministry of Environment data (number of patients per 100,000 people → 2000 = 0.46 person/2025 = 0.46 person/2050 = 0.44 person)	Korea Ministry of Environment (2015), “National climate change adaptation plans”
	Tsutsugamushi	-	Ratio	Build lookup function using the Korea Ministry of Environment data (number of patients per 100,000 people → 2000 = 0.81 person/2025 = 0.81 person/2050 = 0.85 person)	Korea Ministry of Environment (2015), “National climate change adaptation plans”
Risk of climate change	Sea-level rise scenario	-	Km^2^	Use projected value of the submerged area due to sea-level rise in 2100 from research conducted by the Korea Environment Institute (2012)^5^	Korea Environment Institute (2012), “National assessment on sea-level rise impact of Korean coast in the socioeconomic context II”
	Flooding scenario	-	Km^2^	Estimate the linear trend function using annual flood damage data between 2000 and 2014 and extrapolate it to 2050^6^	Ministry of Public Safety and Security (www.safekorea.go.kr),
	Heat wave scenario	-	Day	Build lookup function using both the average number of heat wave days, 7.5 days a year between 1986–2005, and the projected 7.4 additional heat wave days a year in the middle of this century, 2046–2065 (following the RCP 8.5 scenario)	Korea Adaptation Center for Climate Change (www.ccas.kei.re.kr)
	Drought scenario	-	Day	Build lookup function using both the average number of heat wave days, 7.5 days a year between 1986–2005, and the projected 7.4 additional heat wave days a year in the middle of this century, 2046–2065 (following the RCP 8.5 scenario)	Korea Adaptation Center for Climate Change (www.ccas.kei.re.kr)

^1^ Second new town: Pan’gyo, Dongtan, Gimpo, Paju, Gwanggyo, Yangju, Wi’rye, Godeok, and Gumdan.

^2^ Flood damage assessment of the urban area: flood damage assessment of buildings, ships, and public facilities.

^3^ Flood-damaged urban area = flood-damaged area – flood-damaged arable land.

^4^ Food: grain, vegetable, fruit, and livestock.

^5^ According to research conducted by the Korea Environment Institute (2012), the area submerged due to the sea-level rise in 2100 was projected as 3,733 km^2^, and the annual submerged area was calculated by evenly distributing the projected submerged area to years 2006 to 2100. Using this projected value and process, the annual submerged area was calculated by evenly distributing the projected value to years 2000 to 2100 and applying this value to the simulation period, 2000 to 2050.

^6^ The estimated linear trend function was Y = 0.6588X-0.34, and the flooded area in 2050 was projected as 33.2 km^2^.

### Base-run result

#### Overview

**Figure 4:**
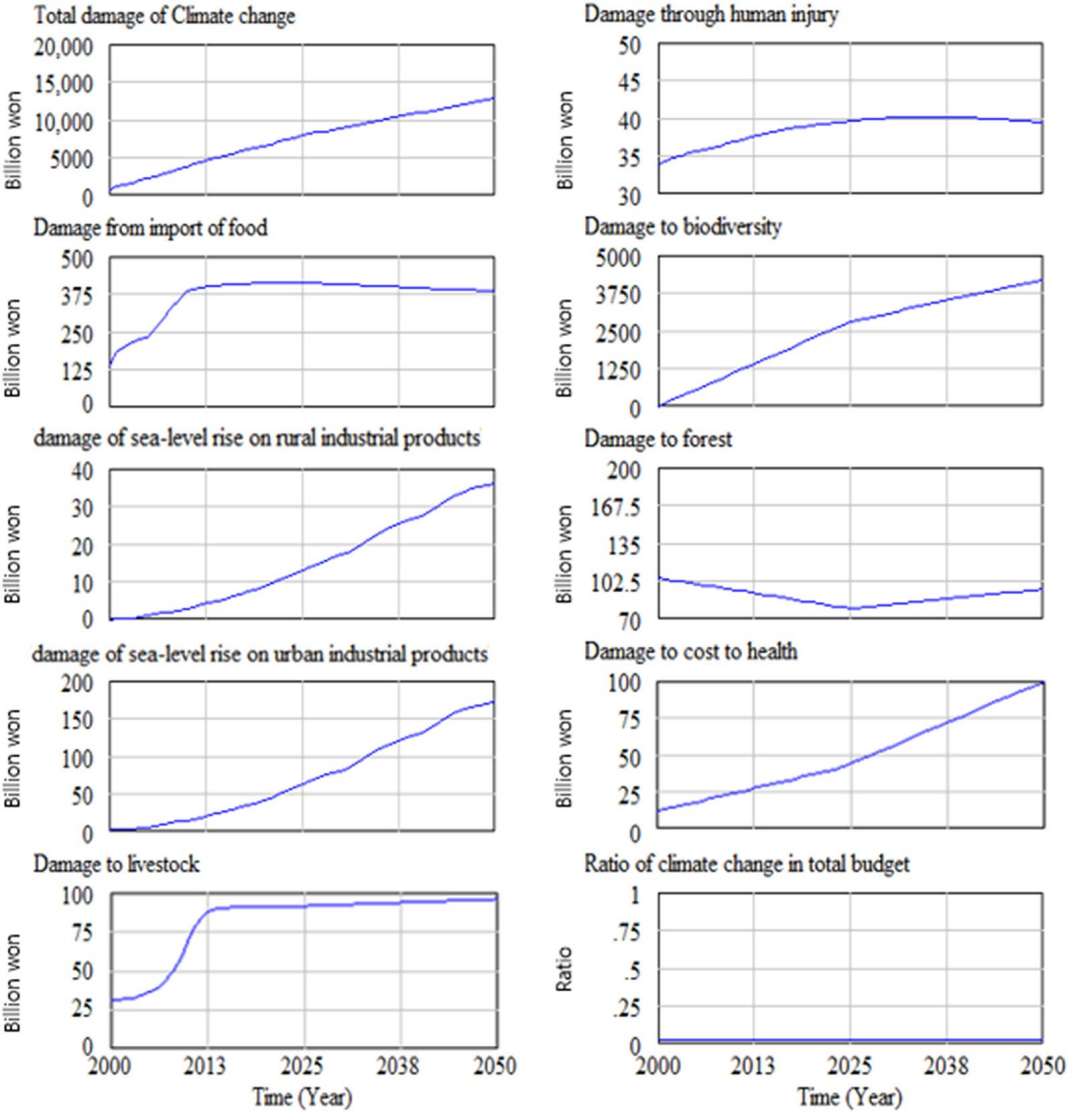
Total damage and damage in each area from climate change. Unit on the vertical axis: billions of Korean won (1 US dollar = approximately 1,100 Korean won).

Our system dynamics simulation starts from the base-run simulation, which presents the result with no policy experimentation. In other words, the base-run simulation provides the result for when the current trend continues with business as usual. A climate change scenario with a representative concentration pathway (RCP) of 8.5 was used for this simulation. Figure 4 shows the climate-change-induced total damage and damage cost in each area of concern with RCP 8.5. The horizontal axis shows time and the vertical axis show the climate-change-induced damage cost.

As Fig. 4 shows, the total cost induced by climate change is continuously increasing in the simulation period—2000 to 2050. Among other areas of concern, the health impact damage cost and cost related to the decrease in biodiversity were higher than that incurred on personal damage due to earlier death and damage to livestock and forests.

#### Urban and rural area damage

**Figure 5:**
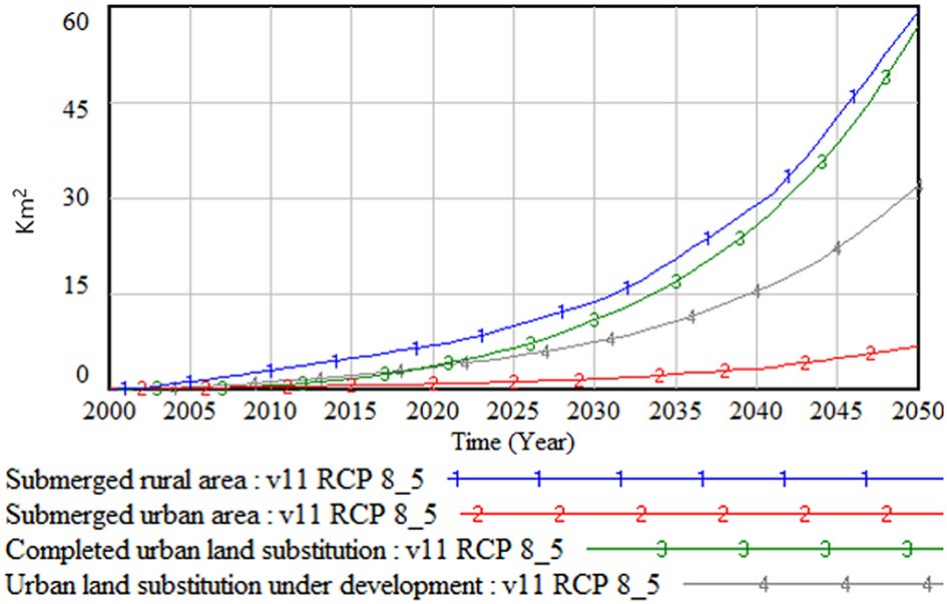
Damaged urban and rural areas.

**Figure 6:**
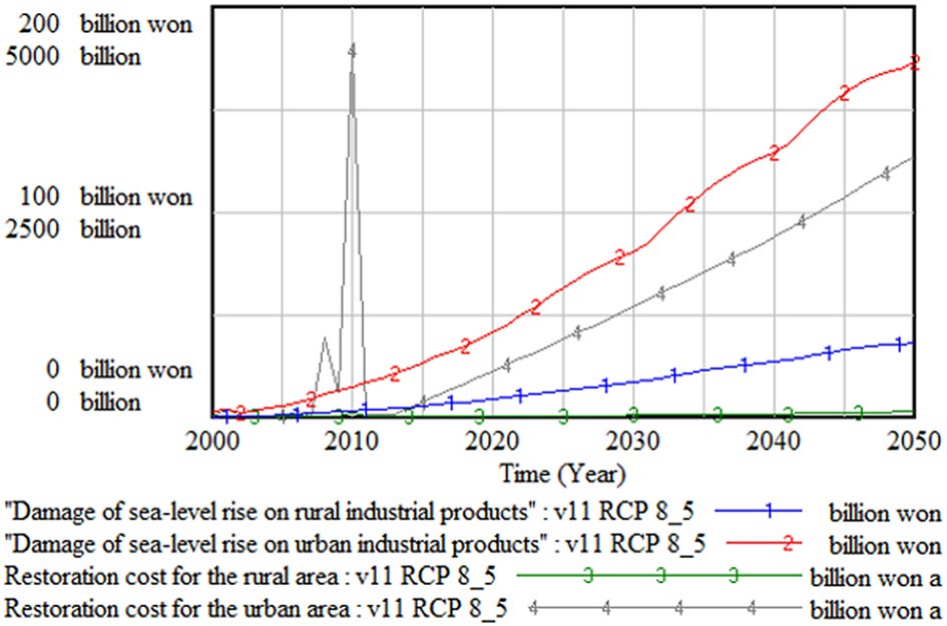
Damage cost in urban and rural areas.

Figures 5 and 6 show the damaged area due to climate change and the damage cost in urban and rural areas, respectively. The simulation result shows that the total damaged rural area is about seven to eight times larger than that the urban counterpart, while the damage cost is about five times higher in the urban area. This result is expected because the output reduction and disaster remediation cost in the urban area are much higher than those in the rural area.

**Figure 7:**
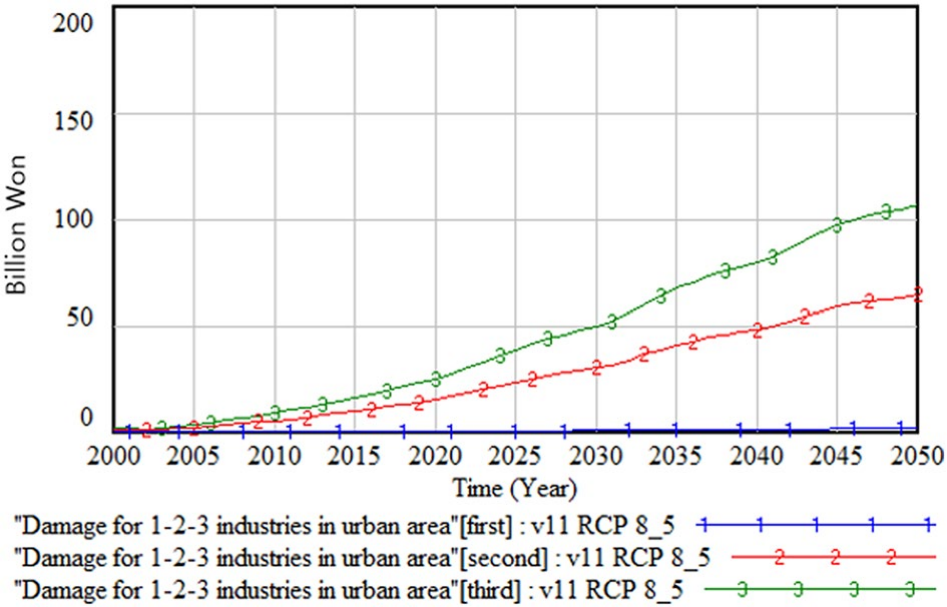
Industry damage in the urban area.

**Figure 8:**
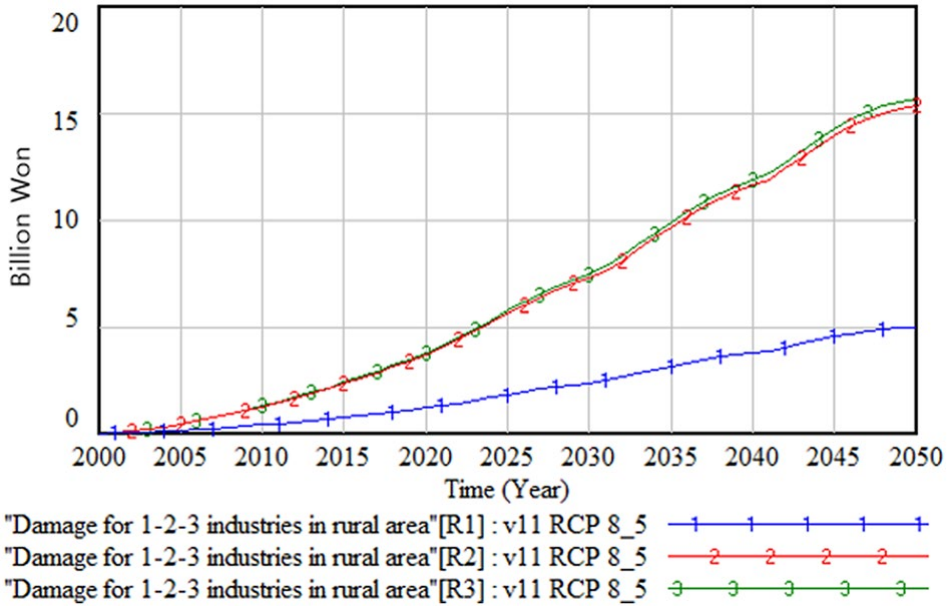
Industry damage in the rural area.

Figures 7 and 8 show the primary, secondary, and tertiary industrial damage costs in the urban and rural areas. In both areas, these costs are highest in the tertiary industry, followed by the secondary and primary industries, even though the scale of damage cost is much less in the rural area than the urban area.

**Figure 9:**
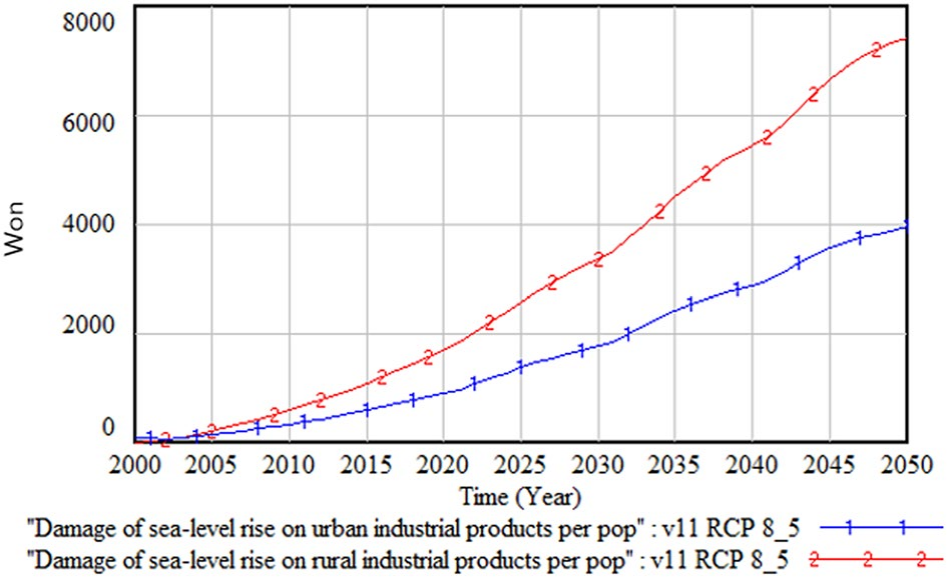
Comparison of industrial output damage between urban and rural areas.

Figure 9 compares the industrial output damage cost per person between urban and rural areas. We observe that the cost in the rural area is about twice of that in the urban area. This result, taken together with Figs. 5 and 6, which show a larger submerged rural area, suggests that rural residents’ perception of climate change damage could be higher than that of urban residents. This implies that rural residents’ perception must be considered when allocating a climate change adaptation budget to these areas. Otherwise, it could trigger a conflict between urban and rural residents regarding fair allocation of budget resources.

#### Food production and population

**Figure 10:**
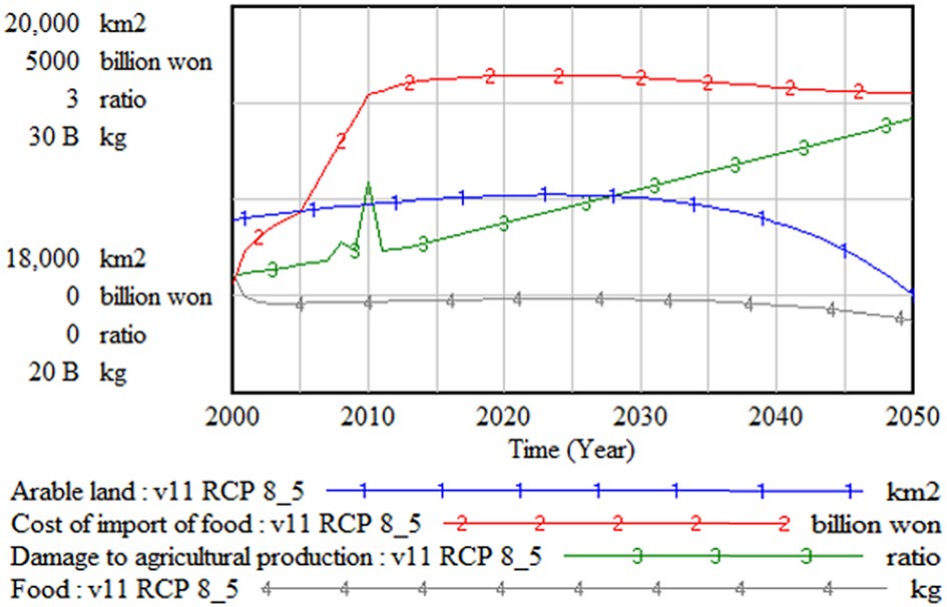
Arable land and food.

**Figure 11:**
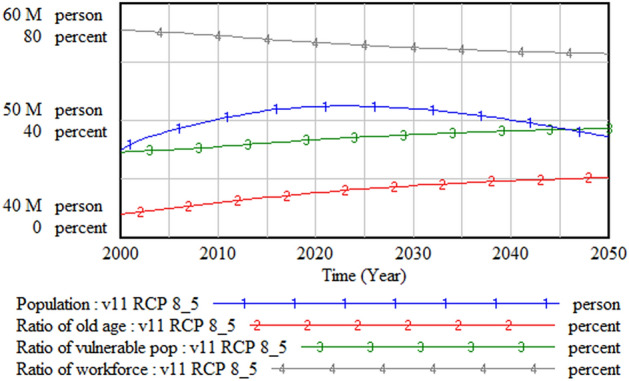
Population and structure.

Figure 10 shows changes in arable land and food production over time. Arable land is continuously decreasing, which decreases food production over time. However, the cost of food import increases rapidly until 2020 and then decreases gradually. The decrease in food import cost is closely related to the population decrease, as shown in Fig. 11.

Figure 11 shows the changes in population structure as well. The ratio of the workforce to the total population is decreasing, while the ratio of the older aged population is increasing. Therefore, the proportion of vulnerable social groups—those aged below 14 and over 65 years—is increasing significantly.

#### Society and the environment

**Figure 12:**
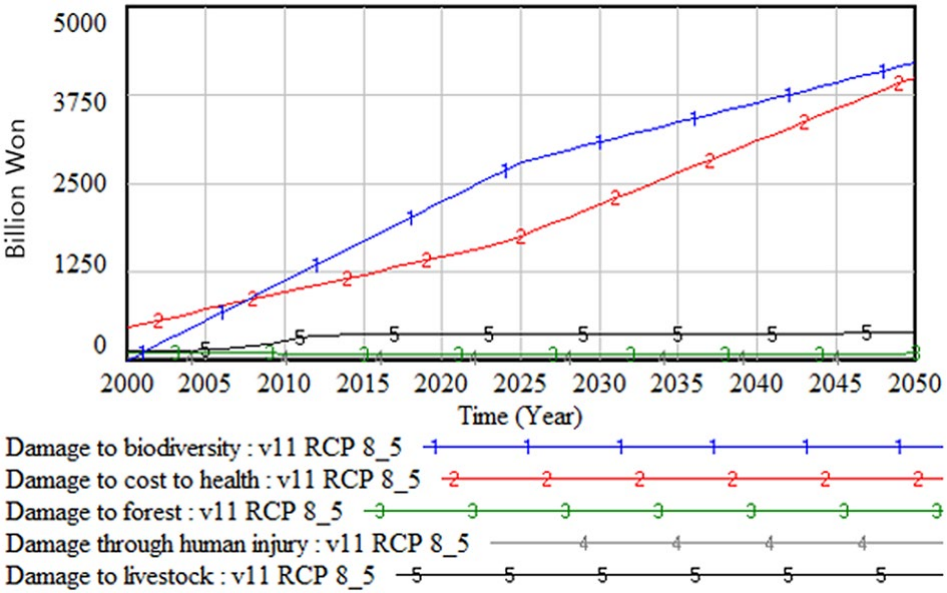
Social and environmental damage.

**Figure 13:**
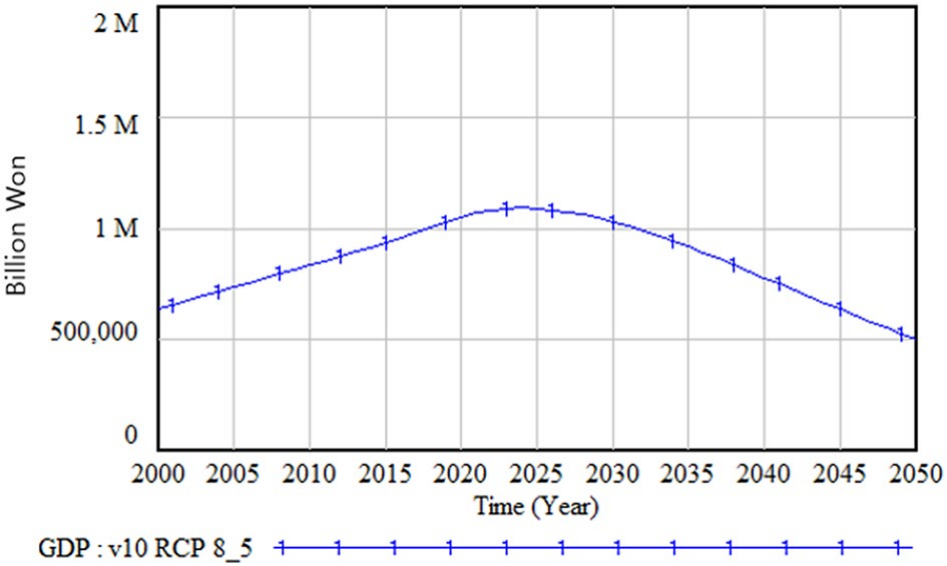
Gross domestic product (GDP) change.

Figure 12 shows the social and environmental damage caused by climate change. Among various types of damage costs, the damage cost due to the decrease in biodiversity is the highest, followed by the damage cost related to health, forests, human injury, and livestock. Figure 13 shows the change in the GDP over time. The GDP peaks between 2020 and 2025 and then begins to decrease. This is because the mounting damage incurred from climate change increasingly diverts the GDP from productive use and investment to damage restoration.

**Figure 14:**
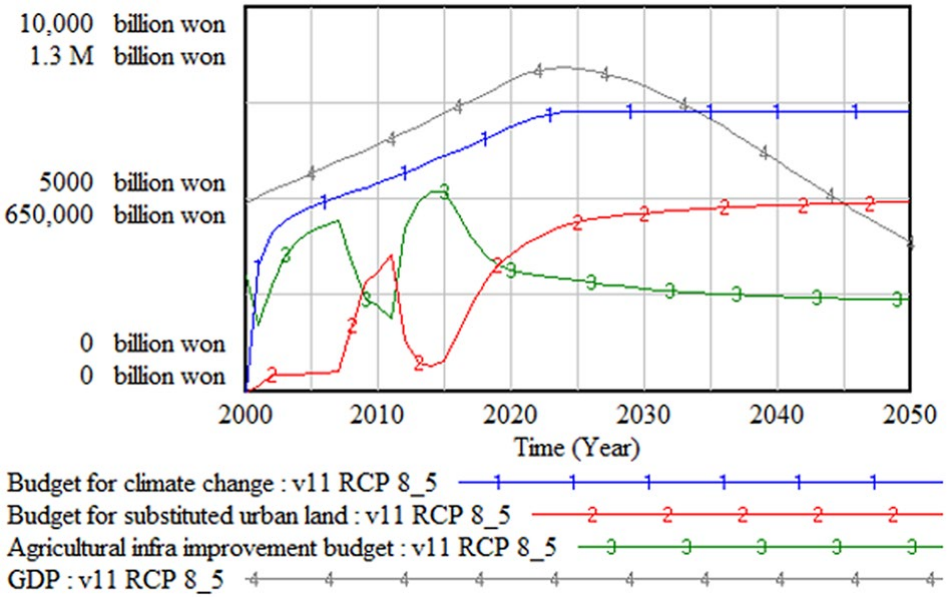
Climate change budget and its use. GDP = gross domestic product.

**Figure 15:**
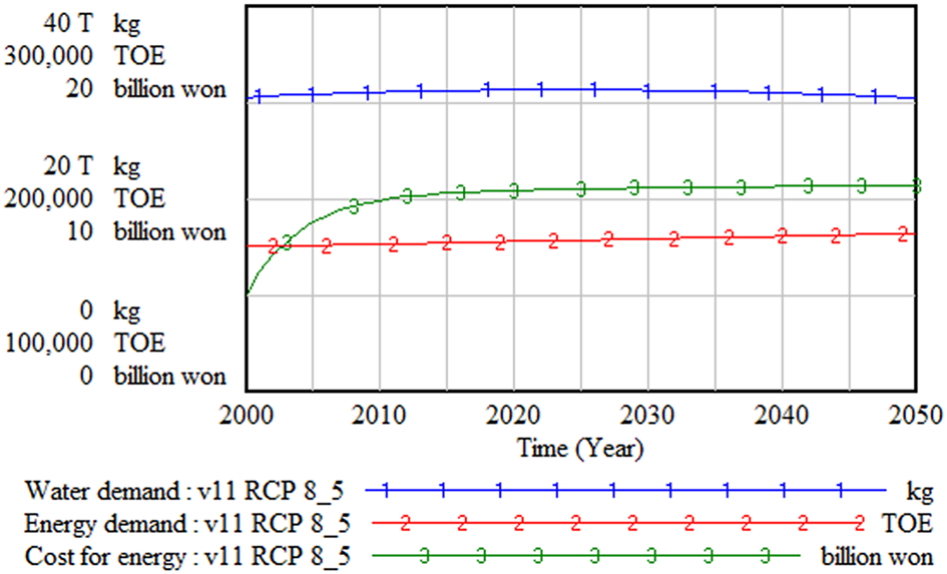
Water, energy demand, and energy cost. TOE = tonne of oil equivalent.

The climate change budget was structured in this model as 3% of the government budget. Since the government budget was modeled as proportional to the GDP, the climate change budget was also affected by the GDP change. It was assumed that the climate change budget is allocated to develop substituted land for submerged urban areas and develop agricultural infrastructure to increase food productivity, and the budgeted amount depends on the degree of urgency. Since the climate change budget accounts for only 0.6% of the GDP, a decrease in the GDP does not significantly affect the trend of the climate change budget, as Fig. 14 shows, and its size can be changed later for policy test simulation. Figure 15 shows the behavior of the water and energy sectors. Water demand decreases with a population decrease, while energy demand and energy cost increase.

In this overview section of base run result, results for climate change impact on urban and rural area, food production, and on society and environment are presented with Fig. 4–15. In summary, the amount of damage caused by climate change has been shown to continue to increase. Industrial production damage in urban and rural areas was simulated greater in urban area than in rural areas but the area and amount of damage per person were greater in rural areas than in urban areas. The damage to agricultural products caused by climate change will continue to increase and the cost of food imports will increase, but as the population decreases, the cost of food imports will decrease from the mid-2020s. The social and environmental damage caused by climate change was analyzed in the order of damage caused by the loss of biodiversity, health damage, forest damage, human life damage and livestock damage.

The results of simulations are largely consistent with the predictions of several climate change-related reports in Korea. According to the Korean Climate Change Assessment Report 2020^26^, flood frequency, flood volume, habitat loss and endangered species, flood damage to coastal and islands areas due to rising sea levels, and pine tree death rates are expected to become more serious in the future. The frequency and scale of forest disasters, especially landslides, are expected to increase. In the agricultural sector, the growth period of rice is expected to shorten, reducing quality and yield, while corn and potatoes are decreasing due to frequent cold weather. The emergence of harmful organisms has also increased, causing the rapid increase of jellyfish and red tide on the coast to occur more frequently and on a larger scale, and damaging coastal fishing^27^. According to government statistics, the damage from natural disasters has reached 7.3 trillion won over the past 10 years (2007–2016), with 162 casualties, and has been on the rise in the long term since 1985^28^. The impact of climate change is significant in biodiversity, health and forestry, indicating the importance of responding to it with long term strategy and plan. In addition, the fact that the total damage in rural areas is smaller than in cities, but the damage area per person and the damage cost per person are larger than in cities indicates that the fair budgetary expenditure between the two regions needs to be carefully considered in climate response policies for conflict prevention.

### Policy test

#### Comparing climate change impact under RCP 8.5 and RCP 4.5

Climate change damage differs with different climate change scenarios. The Representative Concentration Pathways 2.6–8.5 (RCP 2.6, 4.5, 6.0, 8.5) are climate change scenarios used by the IPCC in its 5th report. RCP 8.5 is a scenario in which the concentration of carbon dioxide reaches 940 ppm by the end of the twenty-first century (2070–2099) if the current carbon dioxide emissions continue (Business As Usual) and the global average temperature is expected to rise by 4.8℃. RCP 4.5 is a scenario in which the concentration of carbon dioxide is maintained at 540 ppm and the average temperature is expected to rise by 2.8℃ which is a manageable global temperature increase against climate change impact. The RCP 2.6 scenario is aimed at 420 ppm concentrations, which is already in excess of 420 ppm, making it the best feasible scenario to compare the RCP 4.5 scenario with the prediction of BAU scenario RCP 8.5, which is an important comparison for predicting the impact of policy intervention.

**Figure 16:**
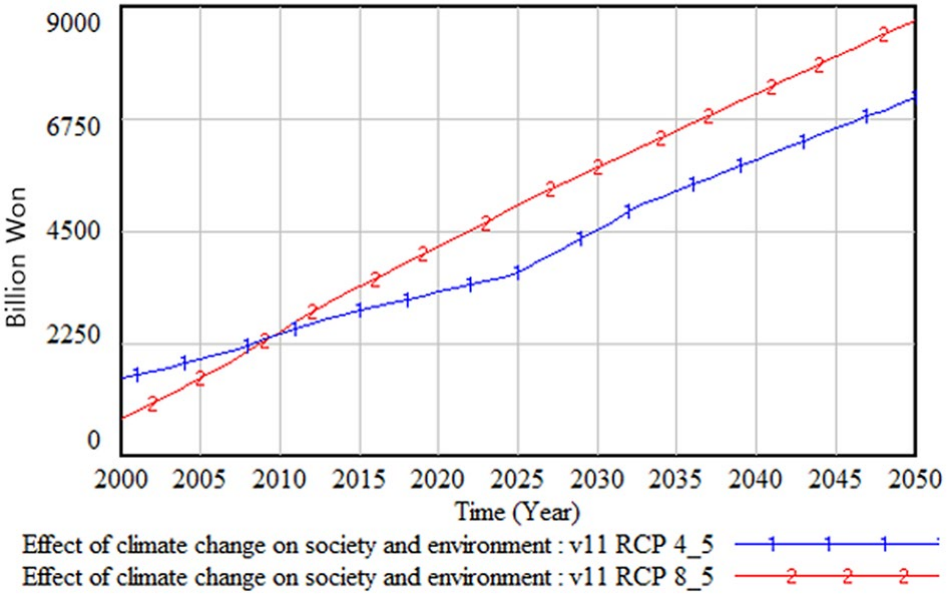
Social and environmental damage under RCP 8.5 vs. RCP 4.5.

**Figure 17:**
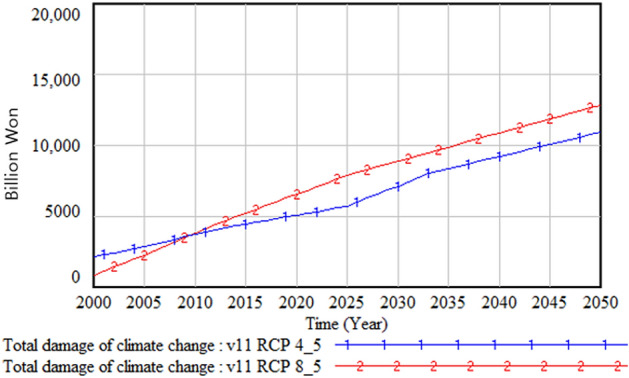
Total damage under RCP 8.5 vs. RCP 4.5.

**Figure 18:**
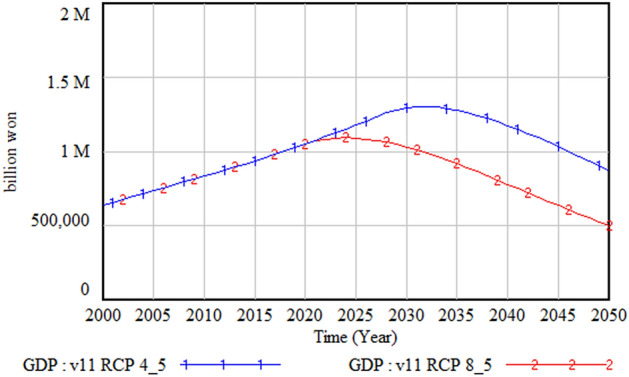
Gross domestic product (GDP) change under RCP 8.5 vs RCP 4.5.

**Figure 19:**
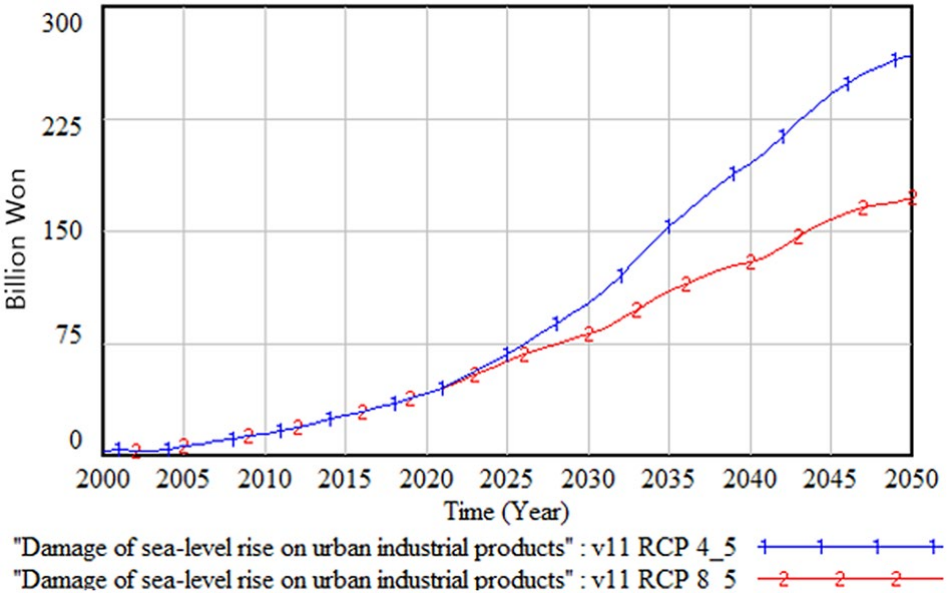
Urban industrial output decrease due to sea-level rise.

**Figure 20:**
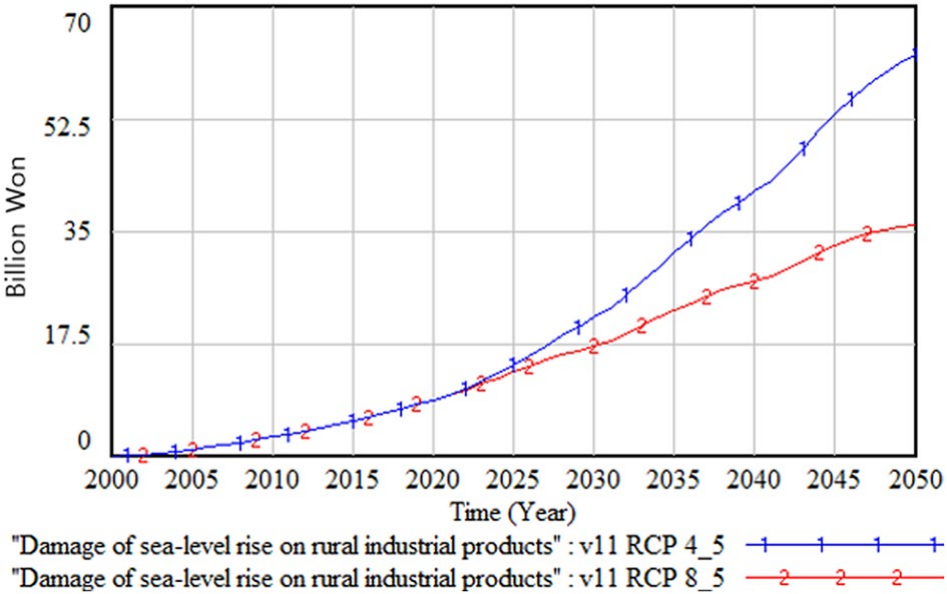
Rural industrial output decrease due to sea-level rise.

**Figure 21:**
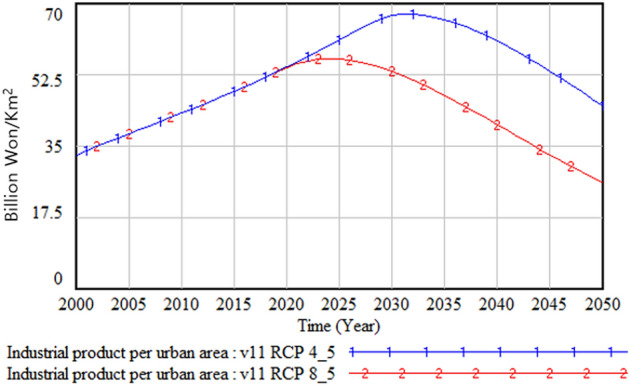
Industrial output per unit of urban area.

The following figures compare climate change damage in several areas of concern under different scenarios—RCP 8.5 and RCP 4.5.Fig. 16 shows the differences in the social and environmental effects of climate change between the RCP 4.5 and 8.5 scenarios. Figures 17 and 18 show the differences in terms of total climate change damage and GDP between the two scenarios. Total climate change damage is greater in the RCP 8.5 scenario (Fig. 16, 17), while GDP (Fig. 18) and industrial output per unit area (Fig. 21) is higher in the RCP 4.5 scenario. When comparing the decrease in industrial output in urban and rural areas due to sea-level rise, Figs. 19 and 20 show that both urban and rural areas see a greater decrease in industrial output under the RCP 4.5 scenario. Figure 21 shows industrial output per unit of urban area is much larger with RCP 4.5 than with RCP8.5 scenario. This is because GDP is higher under the RCP 4.5 scenario, with less damage in the social and environmental area. This lowered climate change damage increases industrial output as well as industrial output damage in both urban and rural areas.

**Figure 22:**
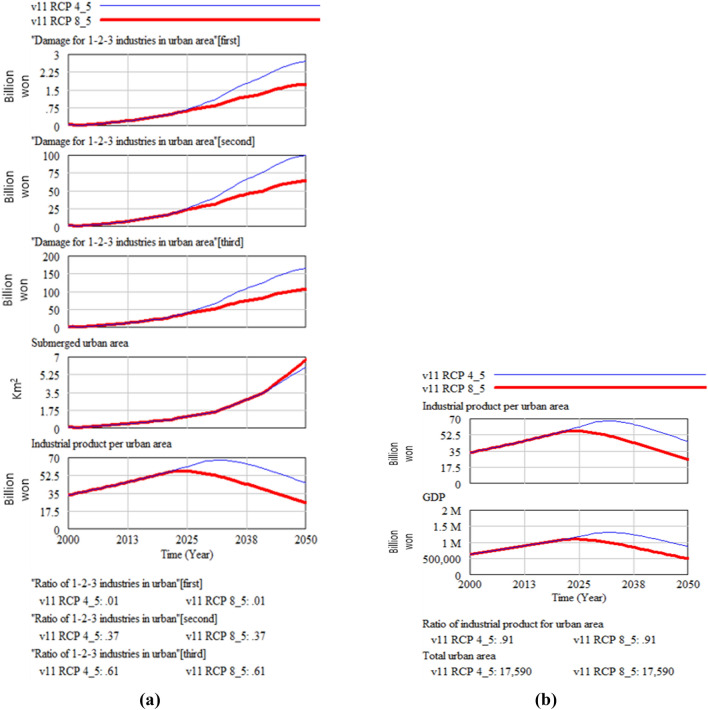
Total urban industrial output, gross domestic product (GDP), and industrial output per unit of urban area.

However, when we consider all the social, environmental, and industrial output reduction damage, the total climate change damage is greater and GDP is smaller in the RCP 8.5 scenario, as shown in Figs. 17 and 18. This result means that under the RCP 8.5 scenario, the social and environmental damage could be greater than the industrial output damage, which could result in industrial output decrease. This implies that the climate change response could be improved by focusing on measures that can maintain industrial output in the short term as well as policy measures that can reduce social and environmental damage in the long term. Figure 22 explains this further.

#### Changes in climate change budget

**Figure 23:**
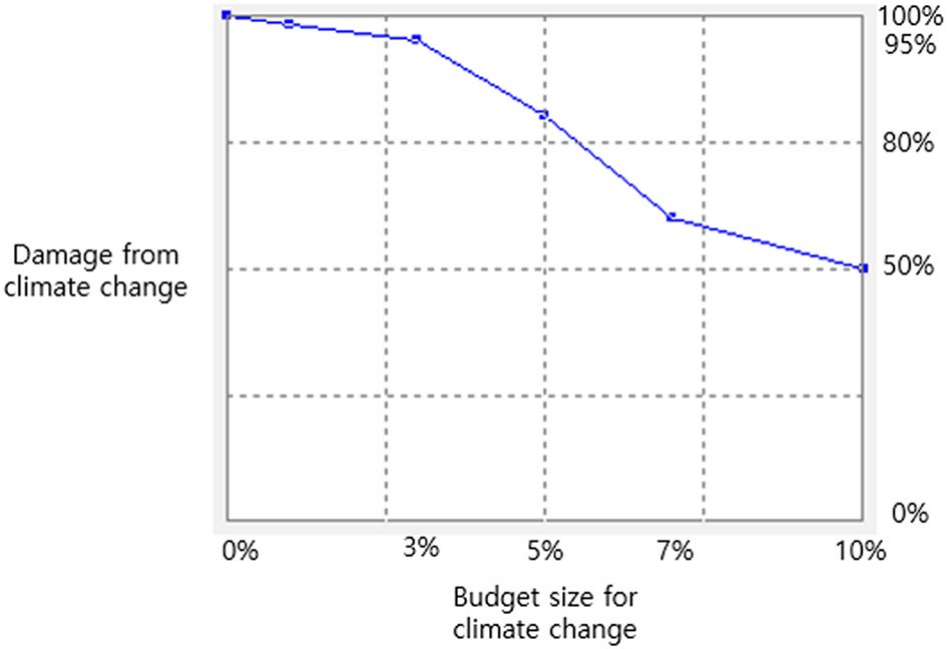
Effect of climate change budget size on climate change damage.

The budget for climate change response can be used to the mitigate the impact of climate change damage by strengthening and preparing infrastructure that can reduce such impact. The budget for climate change in South Korea will be consumed to recover the damages from climate change. Thus, the effects of the climate change budget would be focused on mitigating the size of climate change damages. We used the table function based on the assumption that the climate change budget can reduce the climate-change-induced impact. Figure 23 presents the shape of the function. It was assumed that as the percentage of the climate change budget in the total budget increases, the climate-change-induced impact would reduce, following the function depicted in the figure. We assume that even if the climate change budget is increased to 10% of the total government budget, the climate change damage would not be reduced by more than 50%. The reason that we made this assumption is to avoid the possibility that the government overestimates the effects of the climate change budget. Particularly, even if the government drastically raises the size of the climate change budget, it is generally impossible to recover more than 50% of overall climate change damage in a year. In the figure, the horizontal axis represents the percentage of climate change budget in the total government budget, and the vertical axis shows the percentage change of climate change impact compared with the no climate change budget condition. The relationship between the climate change budget and climate change damage is not always fixed. The shape of the table function can be changed later for policy experiment and sensitivity analysis.

**Figure 24:**
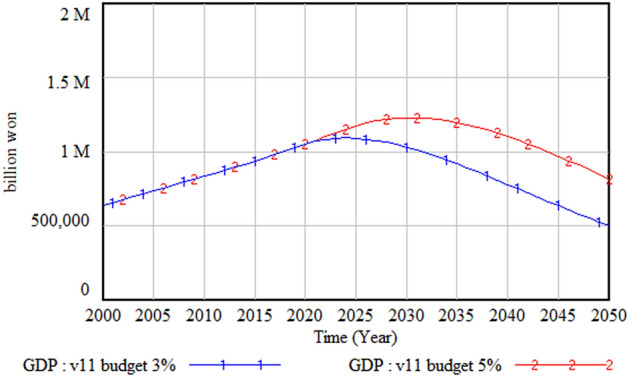
Gross domestic product (GDP) change with climate budget change.

**Figure 25:**
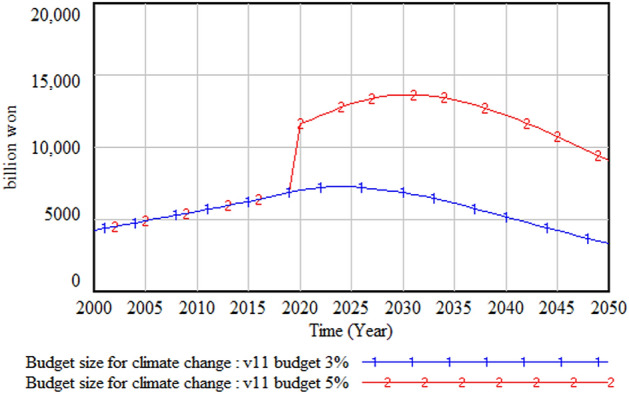
Climate budget change.

**Figure 26:**
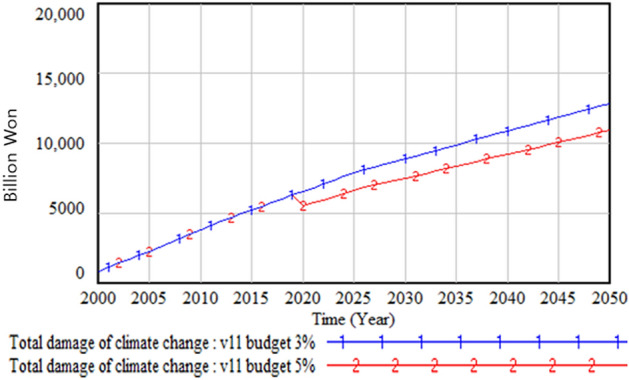
Climate damage with climate budget change.

The policy test results with different climate change budgets—that is, the current 3% and the increased 5% of the total budget size—are shown in Figs. 24, 25 and 26. Figure 24 shows the GDP change when the climate change budget is increased from 3 to 5%. The amount and proportion of the GDP increase due to the increase in the climate change budget is much higher than the climate change budget increase. Figure 25 illustrates the increase in the climate change budget when its share increases from 3 to 5%. It is interesting that a 2% increase in the climate change budget results in a much higher increase in the same budget by 2050, the end of the simulation period. This is because this increase in the climate change budget decreases climate change damage and consequently increases GDP; this, in turn, increases the total amount of the government budget and, accordingly, the climate change budget. This cycle with consecutive results has a virtuous cycle effect, as noted earlier. Figure 26 presents the change in climate change damage when the climate change budget is increased to 5%. With the increased budget, the total climate change damage is reduced from about 13 trillion won to 11 trillion won, and the slope of the climate change damage is decreasing, albeit very slowly.

#### Impact of heat waves on water and energy demand

**Figure 27:**
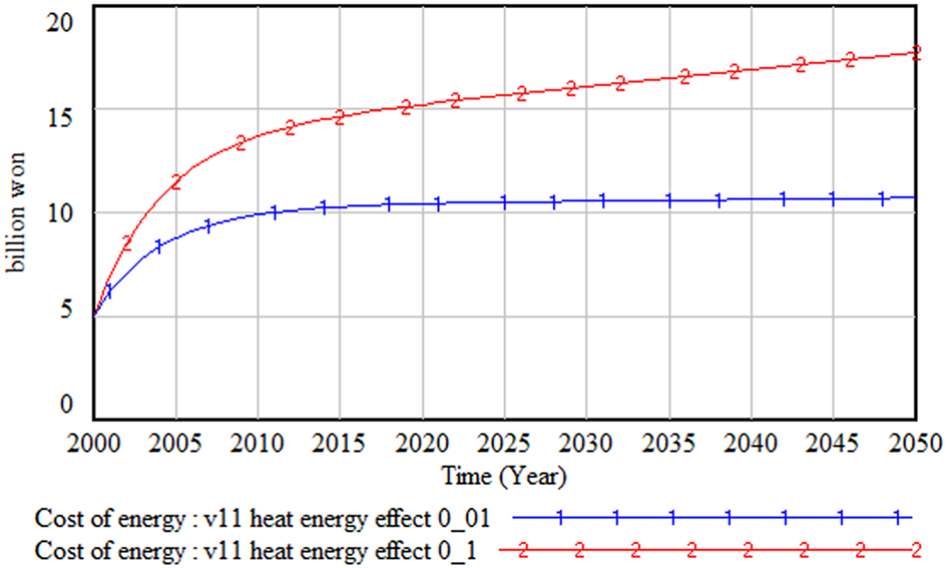
Impact of heat waves on energy cost.

**Figure 28:**
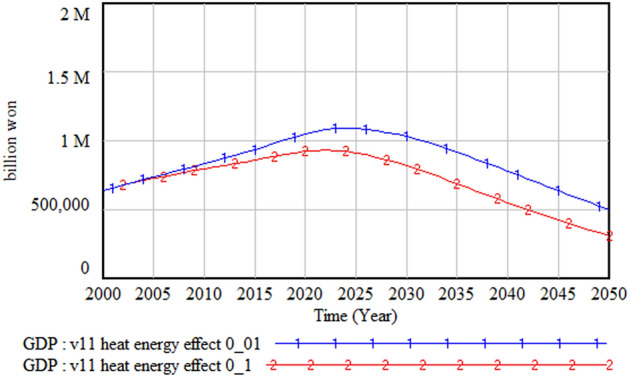
Impact of heat waves on gross domestic product (GDP).

Heat waves affect both water and energy demands. If the increased demands for water and energy are not met, both agricultural and industrial outputs could be seriously damaged, and they could pose a serious threat and cause inconvenience to health and daily life. The impact of heat waves on water and energy demands was simulated by increasing the impact factor of heat wave from 0.01 to 0.1. Figures 27 and 28 respectively show that the heat wave increases energy cost by at least 3.5 times and decreases GDP by more than 200 billion won.

**Figure 29:**
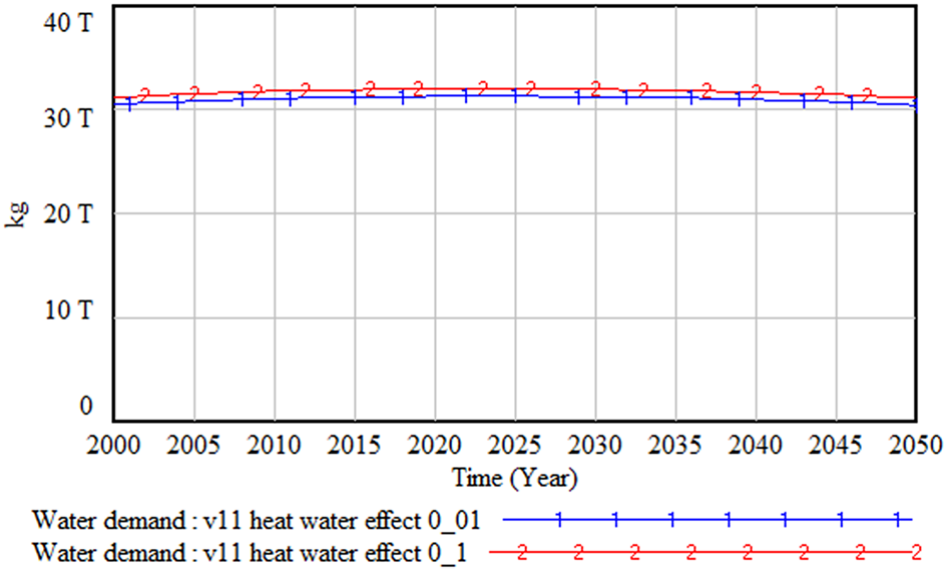
Impact of heat waves on water demand.

However, when we compare the changes in water demand, as shown in Fig. 29, the impact of heat waves on water demand is almost negligible. This is because, as noted earlier, the decreasing population trend reduces the water demand substantially. This result implies that policy measures to tackle heat waves need to focus more on energy than water management.

## Discussion and policy implications

This study used the system dynamics perspective to explain the structure of climate change damage through the CLD. With that, the study constructed a stock-flow diagram and simulated the impacts of climate change-related policies under different climate change scenarios (such as RCP 4.5 and RCP 8.5) in several sectors. The policy implications and the results of the simulation are as follows.

First, most feedback loops in the CLD are positive. The positive loop can generate either a virtuous or vicious cycle depending on the conditions of key variables. For example, with the increase in climate change budget and the decrease in climate change damage, industrial output and GDP would expand. This increased GDP would raise the budget for climate change, which helps to alleviate the impacts of climate change again. As a result, the size of industrial output is growing. This forms a virtuous circle. In contrast, if the climate change budget is decreased and not able to reduce climate change damage in time, the virtuous circle would turn into a vicious circle. Thus, to ensure that the positive feedback structure turns into a virtuous cycle, it is important to create a large climate change budget as early as possible.

Second, regarding the estimated cost of climate-change-induced damage, the cost of losing biodiversity was highest. The costs of impacts on health and foods are following after that. The validation of the estimated cost of losing biodiversity in South Korea is, however, tricky because only limited studies have estimated it. Thus, instead of using them, we employed the world biodiversity value from the UN. We estimated the equivalent proportion of the total biodiversity value based on the share of biospecies in South Korea out of the total biospecies existing globally. Nevertheless, the validity and reliability of this parameter were uncertain. Unfortunately, these are the only available data we can use at this point. If we consider the worldwide trend of increasing attention to the economic value of biological resources, it is hard to ignore the significant damage expected due to the loss of biodiversity. Therefore, a long-term strategy that aims to create the capacity to respond to climate change must consider the protection of biodiversity and biological resources’ stock as well as their sustainability.

Third, when comparing climate change damage, the total damage is larger in the urban area than the rural area. However, the damage per capita and size of the damaged area is higher in the rural area. This implies that the psychological damage might be higher in rural residents. Therefore, when allocating climate change budget or responding to climate damage, the different perceptions of rural and urban residents must be considered to avoid conflict between them.

Fourth, industrial output decreased much more under the RCP 4.5 scenario than under the RCP 8.5 scenario. This indicates that even if worldwide efforts are made to reduce the impact of climate change to that under the RCP 4.5 level, it would be crucial to simultaneously undertake policy measures to mitigate the decline in industrial output.

Lastly, although heat waves affect both energy and water demand, the effect is stronger on energy demand. Therefore, it is necessary to prepare policy measures to improve the energy supply and demand problem. Since an early and large investment to expand supply can increase industrial output and form a virtuous circle in the energy sector, it is important to consider this policy direction seriously. Moreover, for this policy to be effective, measures to reduce the energy supply cost are necessary because, otherwise, a vicious circle might be created.

In conclusion, the impact of climate change on the environment, economy, and society is characterized by a structure dominated by a positive feedback structure. The positive feedback structure becomes a virtuous circle that gets better when it is good and becomes a vicious circle that gets worse when it is bad. Therefore, it is important to develop and implement policies that strengthen the virtuous circle of climate change. In this regard, climate change policies need to expand the virtuous circle through large-scale preemptive investment in the early stages. Efforts for 2050 carbon neutrality or climate change adaptation measures can be approached in the same vein. Larger and earlier investment and policy implementation will lead to a structure in which the virtuous circle is dominating. Investment priorities should be in the area of renewable energy and biodiversity that are expected to benefit the most and to cost the least in the short term as well as in the long term.

Our analysis is based on certain assumptions about the relationship between GDP growth and climate change damage and the damage and the climate change budget. However, the actual relationship between those could be much more complicated than what we assumed in this study. In addition, it could be possible that the impacts of GDP on the climate change budget or the budget on climate change damage do not occur immediately. It could take from few months to several years that the GDP growth raises the size of the climate change budget or the increased climate change budget diminishes the size of climate change damages. This could indicate the limitation of this study that the analysis in this study does not fully reflect the dynamic relationship between different sectors.

The analysis in the study suggests that the increase in the climate change budget corresponding to the GDP growth might be effective to lower the size of climate change damage. However, this does not mean that GDP and climate change produce a virtuous cycle. Instead, the study highlights that the size of the climate change budget should be increased as the GDP grows in order to minimize the size of climate change damage. This is the main positive policy implication of this study.

## Supplementary Information

Supplementary Information.

## References

[1] Sen, A. *Development as freedom.*(Anchor Books Random House Inc., 1999)

[2] Korea Meteorological Administration. *2020 Abnormal Climate Report*(in Korean). (2020).

[3] Forrester, J. W. *World dynamics*. (Wright-Allen Press, Inc., 1971).

[4] Meadows, D. H., Meadows, D. L., Randers, J. & Behrens, W. W. III. *The limit to growth.* (Universe Book, Inc., 1972).

[5] Meadows, D. L., Behrens, W. W. III., Meadows, D. H., Naill, R. F., Randers, J. & Zahn, E. K. O. *The dynamics of growth in a finite world.* (Wright-Allen Press, Inc., 1974).

[6] Meadows, D. H., Meadows, D. L. & Randers, J. *Beyond the limit, global collapse or a sustainable future*. (Earthscan Publications Limited, Inc., 1992).

[7] Meadows, D. L., Randers, J. & Meadows, D. H. *Limits to growth: The 30-year update*. (Chelsea Green Publishing Company, Inc., 2004).

[8] Randers, J. *2052 A global forecast for the next forty years*. (Chelsea Green Publishing, Inc., 2012).

[9] Fiddaman, T. Formulation experiments with a simple climate–economy model. In *Proceedings of the 1995 System Dynamics Conference.* (1995).

[10] Fiddaman T (2002). Exploring policy options with a behavioral climate-economy model. Syst Dynam Rev.

[11] Huerta, J. M., Esquivel-Longoria, M. I. & Arellano-Lara, F. A system dynamics approach to examine climate change impact: The case of the state of Guanajuato, Mexico. In *Proceedings of the 29th International Conference of the System Dynamics.* (2011).

[12] Sterman J, Fiddaman T, Franck T, Jones A, McCauley S, Rice P, Sawin E, Siegel L (2012). Climate interactive: The C-ROADS climate policy model. Syst Dynam Rev.

[13] Song JH, Jeong SJ, Kim KS, Won PJ (2006). System Dynamics Model for Analyzing and Forecasting the National Energy-Economy-Environment(3E) Changes under Levying of Carbon Tax. Korean System Dynamics Review.

[14] Lee, S. E., Choi, D. G. & Park, H. K. Adaptive Management of Water Supply Systems to Deal with Climate Changes: A Gwangdong Dam Case Study. *Journal of Korean Society of Water and Wastewater* **Vol.23. No.5,** 583–598 (in Korean). (2009)

[15] Jang, N. J., Kim, M. K. & Yang, G. S. System Dynamics Application for the Evaluation of Greenhouse Gases Reduction Policy. *Korean System Dynamics Review* **Vol.14. No.4,** 55–68 (in Korean). (2013).

[16] Kim, J. S., Kim, H. Y. & Lee, S. H. Analysis on Inundation Impacts of Sea Level Rise Using System dynamics-GIS Model. *Journal of the Korean Association of Geographic Information Studies* **Vol.18. No.2,** 92–104 (in Korean). (2015).

[17] Chae, Y. R., Choi, S. Y. & Jo, H. J. *Economic analysis of climate change in Korea.* (Korea Environment Institute, 2012).

[18] Moon, T. H. *Sustainable city from the view of system thinking perspective* (In Korean)*.* (Jipmundang, Inc., 2007).

[19] *OECD Environmental Outlook to 2050.*10.1787/9789264122246-en (2012)

[20] Moon, T. H., Kim, D. H., Park, C. S. & Lee, D. S. Policy analysis to reduce climate change-induced risks in urban and rural areas in Korea. *Sustain* **9**: 524. 10.3390/su9040524 (2017)

[21] Richardson, G. P. & Pugh, A. L. III. *Introduction to system dynamics modelling with dynamo*. (The MIT Press, Inc., 1981).

[22] Richmond, B. & Peterson, S. *An introduction to systems thinking.* (High Performance Systems, Inc., 2001).

[23] Kim, D. H., Kim, D. H. & Moon, T. H. *System Dynamics*(in Korean). (Daeyoung Munhwasa, Inc., 1999).

[24] Morecroft, J. *Strategic modelling and business dynamics: A feedback systems approach*. (John Wiley & Sons, Ltd., 2007).

[25] Sterman, J. *Business dynamics, system thinking and modeling for a complex world*. (Irwin McGraw-Hill., 2000).

[26] Ministry of Environment. *Korean Climate Change Assessment Report 2020* (in Korean). (2020).

[27] Korea Meteorological Administration. *Abnormal Climate Report, 10*^*th*^* Anniversary Special Report*(in Korean). (2019).

[28] Ministry of Public Administration and Security. *Statistics for Disaster Announcement, 1985–2018* (in Korean)*.* (2020).

[29] *Climate change 2014* (IPCC, 2014).

