# Supplementary material for: Analyzing climate change impacts on health, energy, water resources, and biodiversity sectors for effective climate change policy in South Korea
Source: Sci Rep. 2021 Sep 16;11:18512. doi: 10.1038/s41598-021-97108-7 (PMC8445984; doi:10.1038/s41598-021-97108-7)
Supplement: Supplementary file 1 — Supplementary Information. [file 41598_2021_97108_MOESM1_ESM.docx]

Analyzing Climate Change Impacts on Health, Energy, Water Resources, and Biodiversity Sectors for Effective Climate Change Policy in South Korea

Tae Hoon Moon ^1^, Yeora Chae ^2,^*, Dong-Sung Lee ^3^ , Dong-Hwan Kim ^1,^* and Hyun-gyu Kim^2^

^1^ Chung-Ang University, College of Social Science, Department of Urban and Real Estate, 84 Heukseok-ro, Dongjak-gu, Seoul, South Korea, 06974; sapphire@cau.ac.kr (T.H.M), baby8803@gmail.com (D.S.L.), sddhkim@cau.ac.kr (D.H.K.).

^2^ Korea Environment Institute, Bldg B, 370 Sicheong-daero, Sejong, Republic of Korea, 30147; yrchae@kei.re.kr (Y.C.) and hgkim@kei.re.kr(H.G.K.).

3. Spatial Information Industry Promotion Agency, 242 Pangyo-ro, Bundang-gu, Seongnam-si, Gyeonggi-do, Korea, 13487; ds.lee@spacen.or.kr(D.S.L.).

***** Correspondence: yrchae@kei.re.kr (Y.C.) and sddhkim@cau.ac.kr (D.H.K.).

Appendix A: Stock and Flow Diagram for the Climate Change Impact Model


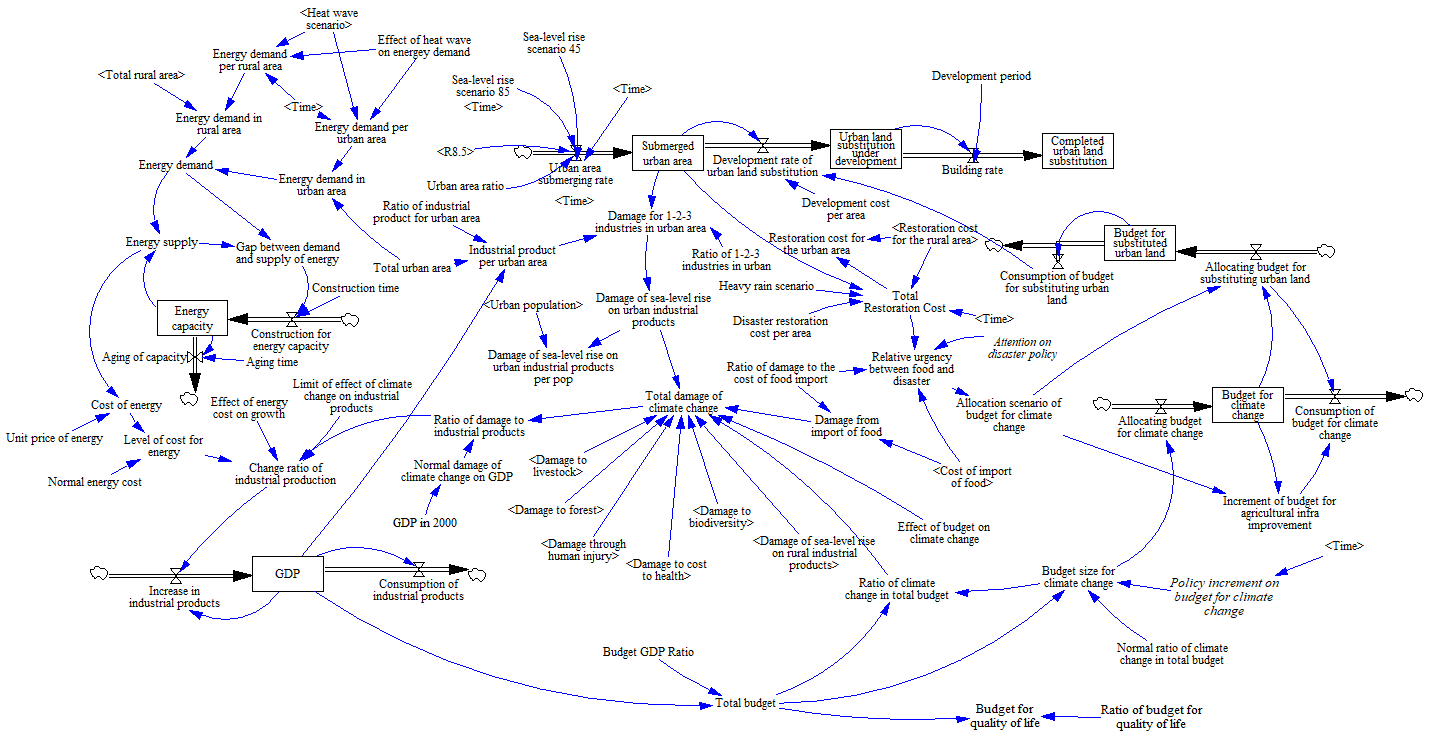


**Fig. A. 1** The urban, energy, industry, and budget sectors. GDP = gross domestic product; 1-2-3 industries = primary, secondary, and tertiary industries.


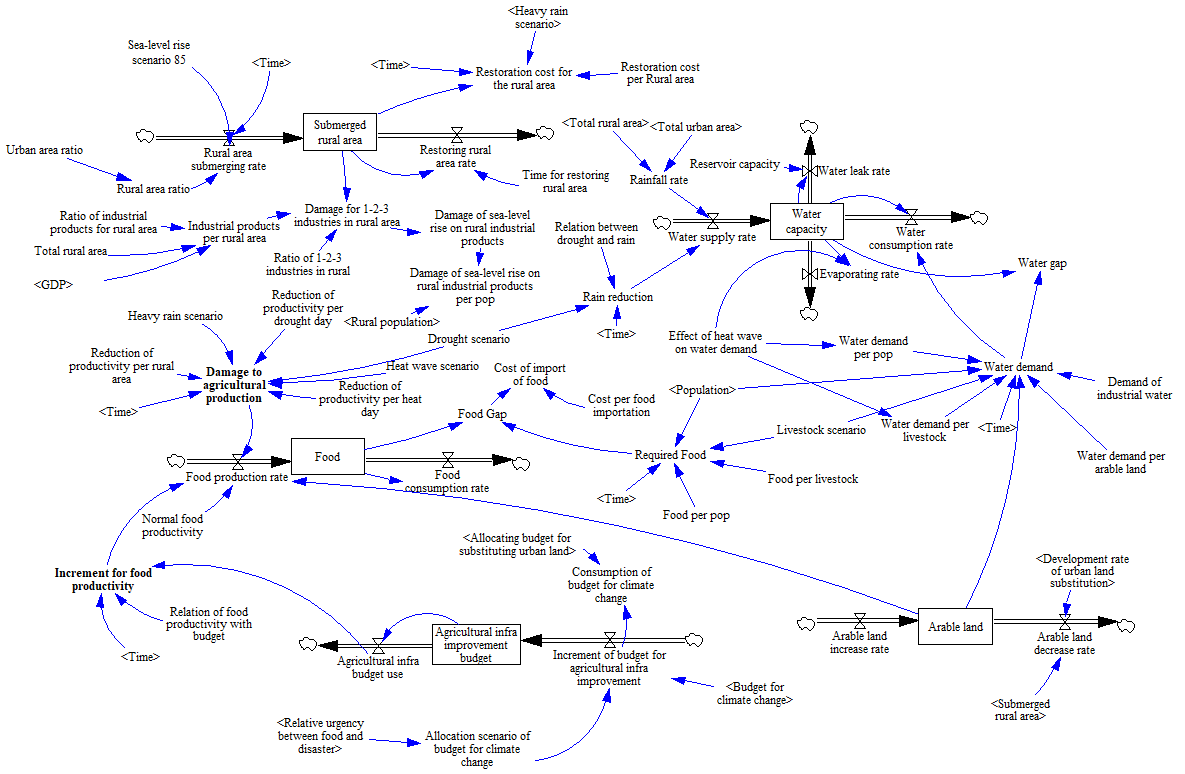


**Fig. A. 2** The rural, food, and water sectors. GDP = gross domestic product; 1-2-3 industries = primary, secondary, and tertiary industries.


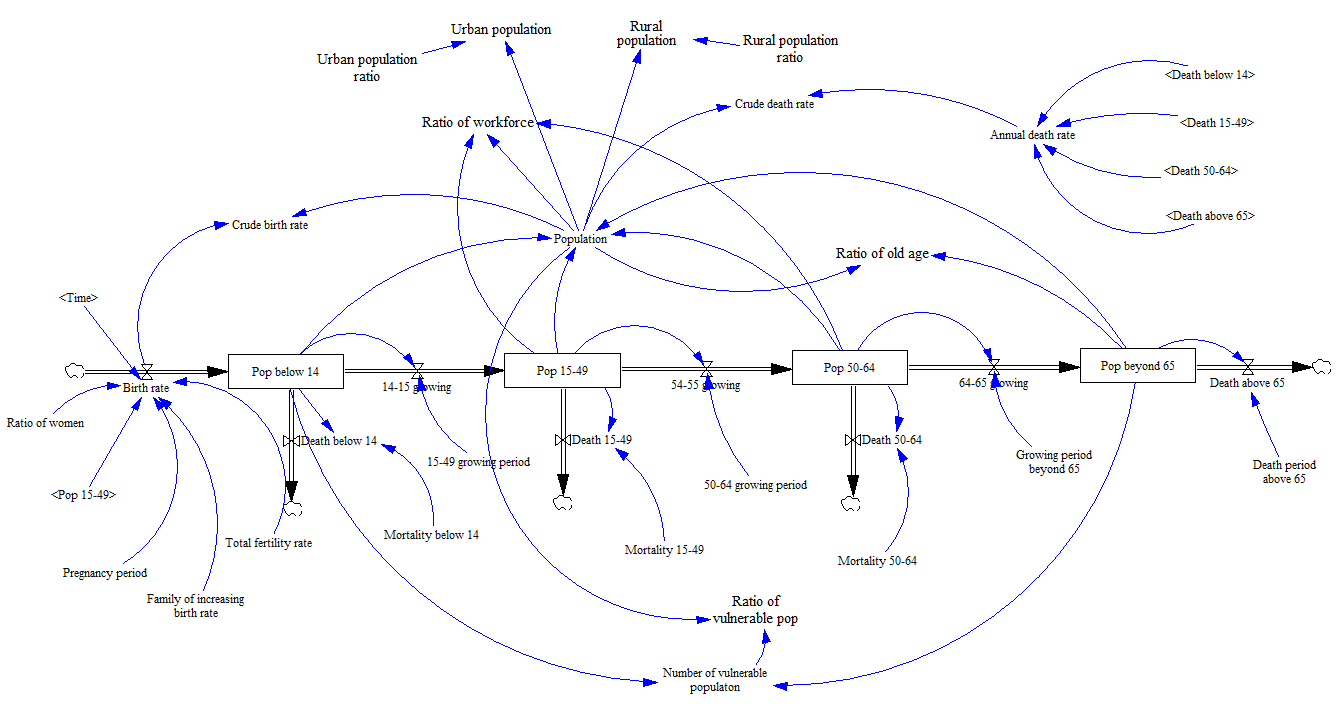


Fig. A. 3 The population sector. The numbers refer to the age groups.


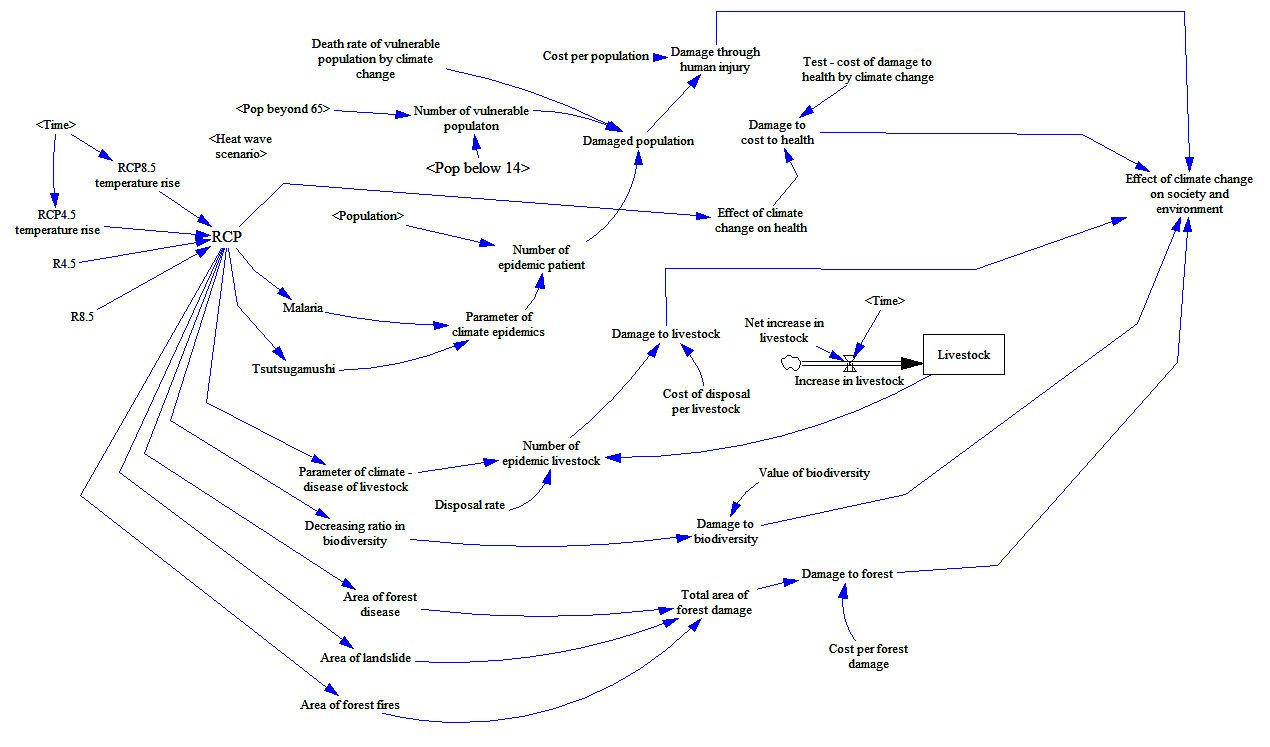


**Fig. A. 4** The social and environmental sectors. RCP = representative concentration pathway. The numbers 14 and 65 refer to the age groups.
